# sumrep: A Summary Statistic Framework for Immune Receptor Repertoire Comparison and Model Validation

**DOI:** 10.3389/fimmu.2019.02533

**Published:** 2019-11-01

**Authors:** Branden J. Olson, Pejvak Moghimi, Chaim A. Schramm, Anna Obraztsova, Duncan Ralph, Jason A. Vander Heiden, Mikhail Shugay, Adrian J. Shepherd, William Lees, Frederick A. Matsen

**Affiliations:** ^1^Fred Hutchinson Cancer Research Center, Seattle, WA, United States; ^2^Department of Statistics, University of Washington, Seattle, WA, United States; ^3^Department of Biological Sciences, Institute of Structural and Molecular Biology, Birkbeck, University of London, London, United Kingdom; ^4^Vaccine Research Center, National Institute of Allergy and Infectious Diseases, National Institutes of Health, Bethesda, MD, United States; ^5^Center of Life Sciences, Skolkovo Institute of Science and Technology, Moscow, Russia; ^6^Genomics of Adaptive Immunity Department, Shemyakin and Ovchinnikov Institute of Bioorganic Chemistry, Moscow, Russia; ^7^Department of Bioinformatics and Computational Biology, Genentech, Inc., South San Francisco, CA, United States; ^8^Department of Molecular Technologies, Pirogov Russian National Research Medical University, Moscow, Russia

**Keywords:** repertoire comparison, model validation, rep-seq, B cell receptor, T cell receptor, summary statistics

## Abstract

The adaptive immune system generates an incredible diversity of antigen receptors for B and T cells to keep dangerous pathogens at bay. The DNA sequences coding for these receptors arise by a complex recombination process followed by a series of productivity-based filters, as well as affinity maturation for B cells, giving considerable diversity to the circulating pool of receptor sequences. Although these datasets hold considerable promise for medical and public health applications, the complex structure of the resulting adaptive immune receptor repertoire sequencing (AIRR-seq) datasets makes analysis difficult. In this paper we introduce sumrep, an R package that efficiently performs a wide variety of repertoire summaries and comparisons, and show how sumrep can be used to perform model validation. We find that summaries vary in their ability to differentiate between datasets, although many are able to distinguish between covariates such as donor, timepoint, and cell type for BCR and TCR repertoires. We show that deletion and insertion lengths resulting from V(D)J recombination tend to be more discriminative characterizations of a repertoire than summaries that describe the amino acid composition of the CDR3 region. We also find that state-of-the-art generative models excel at recapitulating gene usage and recombination statistics in a given experimental repertoire, but struggle to capture many physiochemical properties of real repertoires.

## Introduction

B cells and T cells play critical roles in adaptive immunity through the cooperative identification of, and response to, antigens. The random rearrangement process of the genes that construct B cell receptors (BCRs) and T cell receptors (TCRs) allows for the recognition of a highly diverse set of antigen epitopes. We refer to the set of B and T cell receptors present in an individual's immune system as their immune receptor repertoire; this dynamic repertoire constantly changes over the course of an individual's lifetime due to antigen exposure and the effects of aging.

Although immune receptor repertoires are now accessible for scientific research and medical applications through high-throughput sequencing, it is not necessarily straightforward to gain insight from and to compare these datasets. Indeed, if these datasets are not processed, they are simply a list of DNA sequences. After annotation one can compare gene usage (1–6) and CDR3 sequences. This can be a highly involved task, and so it is common to simply compare the gene usage frequencies and CDR3 length distributions of repertoire (7, 8), leaving the full richness of the CDR3 sequence and potentially interesting aspects of the germline-encoded regions unanalyzed.

An alternative strategy is to transform a repertoire to a more convenient space and compare the transformed quantities according to some distance. For example, several studies reduce a set of nucleotide sequences to *k*mer distributions for classification of immunization status or disease exposure (9–11), where a *k*mer is a nucleotide subsequence of size *k*. These *k*mer distributions can then be compared via sequence-based distances, but still comprise a large space and lose important information about where the *k*mer appears along the sequence. One can perform other dimension reduction techniques such as t-SNE to project repertoires down to an even smaller space (12), but these projections also discard a lot of information and can be difficult to interpret biologically.

While many biologically interpretable summaries such as physiochemical properties exist and have been widely applied (13–16), these are often examined at the sequence level rather than the repertoire level.

We wish to facilitate the use of biologically interpretable summary statistics to capture many different aspects of AIRR-seq data. In addition to enabling comparison of different sequencing datasets, summary statistics can also be used to compare sequencing datasets to probabilistic models to which they have been fitted. Namely, one can use a form of model checking that is common in statistics: after fitting a model to data, one assesses the similarity of the model-generated data to the real data. In this case, we generate a repertoire of sequences from models and compare this collection to a real-data repertoire of sequences via summary statistics.

We are motivated to perform such comparison because these probabilistic models are used as part of inference, and because they are used for inferential tool benchmarking. Such generative models are used to simulate sequences as a “ground truth” for benchmarking inferential software (17–19), and thus the accuracy of such benchmarks depends on the realism of the generated sequences. Simulation tools can also be used to generate a null distribution used to test for a specific effect, such as natural selection (20).

Currently, there are no unified packages dedicated to the task of calculating and comparing summary statistics for AIRR-seq datasets. While the Immcantation framework (which includes the shazam and alakazam R packages) contains many summary functions for AIRR-seq data (21), it does not have general functionality for retrieving, comparing, and plotting these summaries. Many summaries of interest are implemented in one package or another, but differences in functionality and data structures make it troublesome to compute and compare summaries across packages. Some summaries of interest, such as the distribution of positional distances between mutations, are not readily implemented in any package.

In this paper, we gather dozens of meaningful summary statistics on repertoires, derive efficient and robust summary implementations, and identify appropriate comparison methods for each summary. We present sumrep, an R package that computes these summary distributions for AIRR-seq datasets and performs repertoire comparisons based on these summaries. We investigate the effectiveness of various summary statistics in distinguishing between different experimental repertoires as well as between simulated and experimental data. We show that many summaries differentiate between various covariates by which the datasets are stratified. Further, we demonstrate how sumrep can be used for model validation through case studies of two state-of-the-art repertoire simulation tools: IGoR (19) applied to TRB sequences, and partis (17, 22) applied to IGH sequences.

## Results

### Implementation

The full sumrep package along with the following analyses can be found at https://github.com/matsengrp/sumrep. It supports the IGH, IGK, and IGL loci for BCR datasets, and the TRA, TRB, TRD, and TRG loci for TCR datasets. It is open-source, unit-tested, and extensively documented, and uses default dataset fields and definitions that comply with the Adaptive Immune Receptor Repertoire (AIRR) Community Rearrangement schema (23). A reproducible installation procedure of sumrep is available using Docker (24).

Table 1 lists the summary statistics currently supported by sumrep, and includes the default assumed degree of annotation, clustering, and phylogenetic inference for each summary. The first group of statistics only requires the input or query sequences to be aligned to their inferred germline sequences (e.g., IMGT-aligned) and constrained to the variable region; this coincides with the presence of the sequence_alignment and germline_alignment fields in the AIRR schema (we note that some of these statistics, such as GC content, do not require an alignment in principle. However, we wished to encourage meaningful analyses and comparisons with our software, and thus require an alignment to avoid accidental comparison of non-corresponding sequence regions). The second group requires standard sequence annotations, such as inferred germline ancestor sequences for Ig loci, germline gene assignments, and indel statistics. The third group requires clonal family cluster assignments. The fourth group requires a inferred phylogeny for each clonal family of an Ig dataset. sumrep itself does not perform any annotation, clustering, or phylogenetic inference, but rather assumes such metadata are present in the given dataset; in principle, one can use any tool which performs these tasks as expected.

**Table 1:** Currently supported summary statistics grouped by their respective degrees of assumed post-processing.

**Summary statistic**	**Annotations**	**Clustering**	**Phylogeny**	**Implementation**
Pairwise distance distribution	No	No	No	stringdist (25)
*k*th nearest neighbor distribution	No	No	No	stringdist
GC-content distribution	No	No	No	ape (26)
Hotspot motif count distribution	No	No	No	Biostrings (27)
Coldspot motif count distribution	No	No	No	Biostrings (27)
CDR3 length distribution	Yes	No	No	Tool-provided
Joint distribution of germline gene use	Yes	No	No	sumrep
Pairwise CDR3 distance distribution	Yes	No	No	stringdist
Atchley factor distributions	Yes	No	No	HDMD (28)
Kidera factor distributions	Yes	No	No	Peptides (28)
Aliphatic index distribution	Yes	No	No	Peptides
G.R.A.V.Y. index distribution	Yes	No	No	alakazam (21)
Polarity distribution	Yes	No	No	alakazam
Charge distribution	Yes	No	No	alakazam
Basicity distribution	Yes	No	No	alakazam
Acidity distribution	Yes	No	No	alakazam
Aromaticity distribution	Yes	No	No	alakazam
Bulkiness distribution	Yes	No	No	alakazam
Per-gene substitution rate	Yes	No	No	Tool-provided + sumrep
Per-gene-per-position substitution rate	Yes	No	No	Tool-provided + sumrep
Per-base substitution model	Yes	No	No	shazam (21)
Per-base mutability model	Yes	No	No	shazam
Positional distance between mutations distribution	Yes	No	No	sumrep
Distance from germline to sequence distribution	Yes	No	No	stringdist
V gene 3′ deletion length distribution	Yes	No	No	Tool-provided
V gene 5′ deletion length distribution	Yes	No	No	Tool-provided
D gene 3′ deletion length distribution	Yes	No	No	Tool-provided
D gene 5′ deletion length distribution	Yes	No	No	Tool-provided
J gene 3′ deletion length distribution	Yes	No	No	Tool-provided
J gene 5′ deletion length distribution	Yes	No	No	Tool-provided
VD (or VJ) insertion length distribution	Yes	No	No	Tool-provided
DJ insertion length distribution	Yes	No	No	Tool-provided
VD (or VJ) insertion transition matrix	Yes	No	No	sumrep
DJ insertion transition matrix	Yes	No	No	sumrep
V/J in-frame percentage	Yes	No	No	Tool-provided + sumrep
Cluster size distribution	Yes	Yes	No	Custom
Hill numbers (diversity indices)	Yes	Yes	No	alakazam
Selection estimates (using the BASELINe method)	Yes	Yes	No	shazam
Sackin index distribution	Yes	Yes	Yes	CollessLike (29)
Colless-like index distribution	Yes	Yes	Yes	CollessLike
Cophenetic index distribution	Yes	Yes	Yes	CollessLike

*Annotation denotes whether annotation of the V(D)J germline segment is required, Clustering denotes whether clonal clustering is required, and Phylogeny denotes whether lineage tree inference is required. “Tool-provided” means that the summary can be directly computed from the output of an annotation tool; for example, the CDR3 length distribution is exactly the frequencies of values in the junction column of the annotated dataset. Per-gene substitution rate is defined to be the number of observed mutations in sequences assigned to that gene, in the segment of the sequence assigned to that gene's region, divided by the length of the segment. Per-gene-per-position substitution rate is similarly defined, but separately computed for each position in the sequence*.

sumrep contains many types of summaries, including nucleotide sequence-level summaries (pairwise distances, hotspot motif counts, etc.), rearrangement summaries like insertion and deletion lengths, and many physiochemical properties applicable to the amino acid sequences of particular receptor regions. The Atchley factors are a set of five numerical descriptions of amino acids derived using a statistical technique called factor analysis from a larger pool of 494 descriptors of amino acid biochemical properties (30). The Kidera factors are a similarly-constructed set of ten numerical descriptions of amino acids, which were derived using dimension reduction techniques (31). sumrep also includes summaries to be applied at the clonal family level (e.g., cluster size distribution) and the phylogenetic level in the case of BCR sequences (e.g., Sackin index distribution).

sumrep makes it easy to compare summary statistics between two repertoires by equipping each summary with an appropriate divergence, or measure of dissimilarity, between instances of a summary. For example, the getCDR3LengthDistribution function returns a vector of each sequence's CDR3 length, and the corresponding compareCDR3LengthDistributions function takes two repertoires and returns a numerical summary of the dissimilarity between these two length distributions. The comparison method depends on the summary, which is discussed further in the Methods section. sumrep also includes a compareRepertoires function which takes two repertoires and returns as many summary comparisons as befit the data.

**Figure 1:**
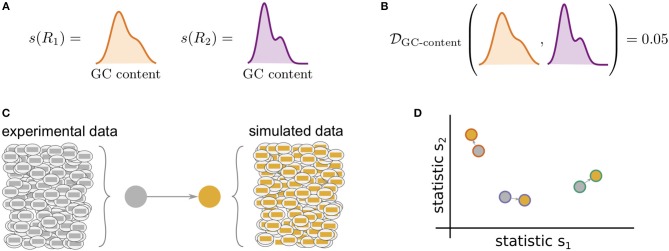
Cartoon of our summary statistic and divergence framework, and how this can be applied to validation of repertoire simulators. Steps **(A,B)** can be applied to compare arbitrary datasets, while **(C,D)** show how sumrep can be used for model validation. **(A)** Most summary statistics *s*, e.g., GC content, yield a distribution of values when applied to each of the sequences in a given repertoire *R*. **(B)** We can compare summary distributions using a statistical divergence D, which takes two distributions and outputs a nonnegative scalar. **(C)** For a given experimental dataset, we use simulator tools to generate a corresponding set of synthetic sequences. **(D)** We can compute many summaries of these repertoires yielding distributions for comparison.

Figure 1 illustrates the general framework of comparing summary statistics between two repertoires *R*_1_ and *R*_2_. A given summary *s* is applied separately to *R*_1_ and *R*_2_, which for most summaries yields a distribution of values (Figure 1A). These two resultant distributions can be compared using a divergence D that is tailored to the nature of *s* (Figure 1B). We use Jenson-Shannon (JS) divergence to compare scalar distributions (e.g., GC content, CDR3 length), which is a symmetrized version of KL-divergence, a weighted average log-ratio of frequencies widely-used in statistics and machine learning. We use the similarly popular ℓ_1_ divergence to compare categorical distributions (e.g., gene call frequencies, amino acid frequencies), which is a sum of absolute differences of counts.

We have designed sumrep to efficiently approximate computationally intensive summaries. When the target summary is a distribution, we can gain efficiency by repeatedly subsampling from the distribution until our estimate has stabilized. The result is an approximation to the full distribution; by introducing slight levels of noise, we can gain very substantial runtime performance improvements for large datasets. This in turn allows fast, accurate divergence estimates between dataset summaries. We outline a generic distribution approximation algorithm as well as a modified version for the nearest neighbor distance distribution in the Methods section, and conduct extensive empirical validation of these algorithms in Appendices A, B.

sumrep additionally contains a plotting function for each univariate summary distribution. For example, the getCDR3LengthDistribution comes with a companion plotting function called plotCDR3LengthDistribution. sumrep also includes a master plotting function, plotUnivariateDistributions, which shows a gridded figure of all univariate distribution plots relevant to the locus in question which can be computed from the input dataset. Currently, these plotting functions support frequency polygons and empirical cumulative distribution functions (ECDFs). Examples of these plots can be found throughout later sections of this report.

### Application of Summary Statistics to Experimental Data

To examine the ability of various summary statistics to distinguish among real repertoires, we applied sumrep to TCR and BCR datasets performed a multidimensional scaling (MDS) analysis of summary divergences. In particular, we computed divergences of each summary between each pair of repertoires, stratified by covariates such as individual, timepoint, and cell subset to form a dissimilarity matrix. We then mapped these dissimilarity matrices to an abstract Cartesian space using MDS.

**Figure 2:**
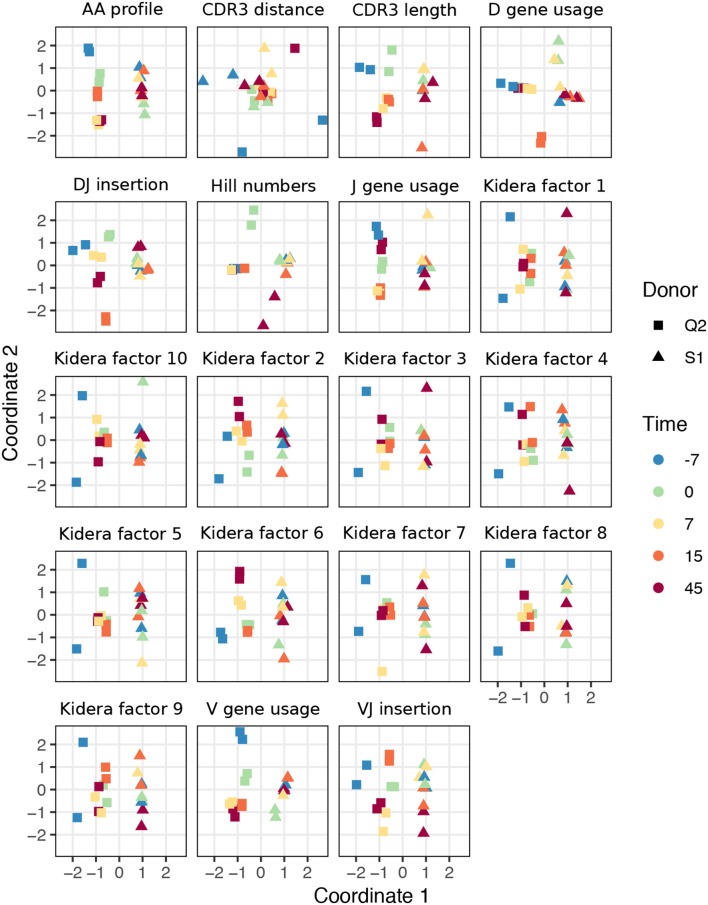
Plots of summary divergence MDS coordinates for data from Pogorelyy et al. (32), grouped by donor and timepoint.

For TCR repertoires, we used datasets from two individuals and five timepoints post-vaccination, with two replicate per donor-timepoint value, from Pogorelyy et al. (32). Figure 2 displays plots of the first two coordinates of each replicate grouped by donor and timepoint. We see that for almost all summaries, these points cluster according to donor identity, with the CDR3 pairwise distance distribution being the only summary that does not decisively cluster by donor. Many summaries additionally cluster according to timepoint in the second dimension, although the tightness of clustering varies by summary, with some summaries (e.g., DJ insertion length distribution) being tightly clustered by a given donor/timepoint value and some summaries (e.g., Kidera factor 4) not obviously clustering by donor/timepoint. Moreover, the D gene usage distribution for each individual splits into two distinct groups which do not correlate with timepoint, though the import of this is more difficult to assess. Although these patterns would require further exploration in a particular research context, these sumrep divergences show interesting patterns when TCR datasets are stratified by covariates.

**Figure 3:**
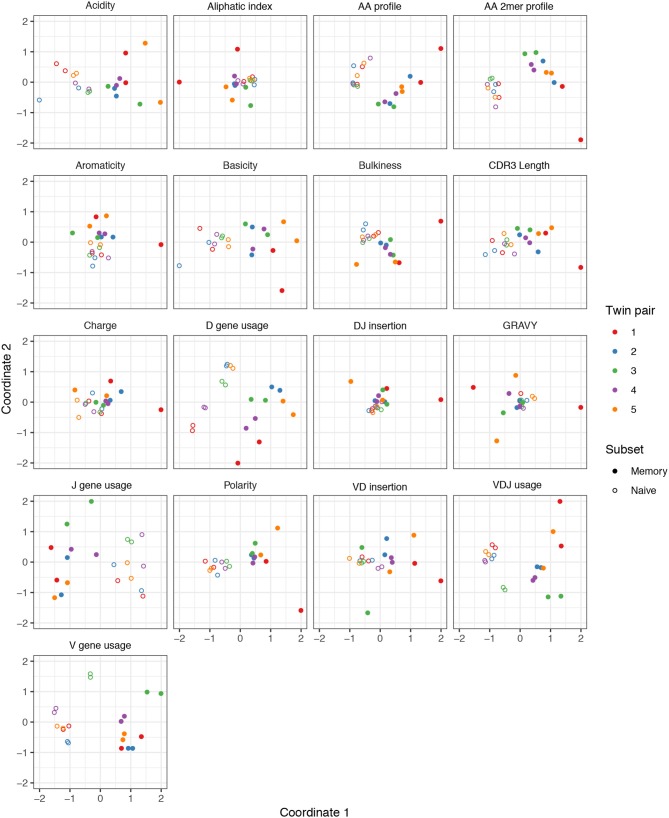
Plots of summary divergence MDS coordinates for data from Rubelt et al. (33), grouped by twin pair identity and cell type (memory vs. naive).

We performed a similar MDS analysis of summary divergences of BCR repertoires stratified by covariate, using data from Rubelt et al. (33). We computed divergences of each summary between each pair of a collection of datasets stratified by five pairs of twins as well as B cell classification as memory or naive to form a dissimilarity matrix. We then mapped these dissimilarity matrices to an abstract Cartesian space using MDS. Figure 3 displays plots of the first two coordinates of each donor grouped by twin pair identity and cell type. We see that for each summary, points can be separated according to cell subset, with some summaries (e.g., V gene usage, AA frequencies, acidity) clustering more tightly among cell subset, and others (e.g., GRAVY index, DJ insertion length) clustering more loosely. In addition, the naive repertoires appear to be more tightly clustered than the memory repertoires for each summary. Finally, for the gene usage statistics, there is a strong tendency for twins to have higher similarity than unrelated donors, although this tendency is not consistently observed for other statistics. For example, points for the amino acid 2mer frequency distribution divergences tend to have high similarity between twins, but the GRAVY index distribution divergences do not. Thus, there seem to also be interesting dynamics underlying sumrep divergences when BCR datasets are stratified by covariates, and the observed patterns merit further investigation.

### Ranking Summary Statistic Informativeness

Due to the large number of summary statistics supported by sumrep, many of which are correlated, we sought an approach to identify a set of maximally-informative statistics that provide complimentary information to one another. To address this, we employed a lasso multinomial regression treating certain sequence-level summaries as covariates and dataset identity as the response. The basic idea is that this regression method cuts out all but a few predictor variables to find a smaller collection of informative summary statistics, as a coefficient is “allowed” to be nonzero only when the lasso deems it a relatively meaningful predictor. As the regularization parameter λ is decreased, more and more coefficients become nonzero, leading to a natural ordering of summaries as the order in which their coefficient “branches off” from zero. Then a resultant maximally-informative set of *k* summaries is the set of summaries with the *k* best ranks. We formalize this approach in the Methods section (Algorithm 3).

One caveat to this approach is that we can only use sequence-level summary statistics as covariates in order to have a well-defined regression procedure. However, the majority of summaries considered in this report are applied at the sequence level. Thus, between the subset of informative sequence-level statistics and the remaining non-sequence-level statistics, we arrive at a considerably smaller set. Besides non-sequence-level summaries, we also omit Kidera Factors and Atchley factors from our analyses as these sets of statistics are orthogonal by construction according to particular measures of amino acid composition in their respective original contexts. This also leads to a much smaller design matrix and a substantially decreased runtime.

**Figure 4:**
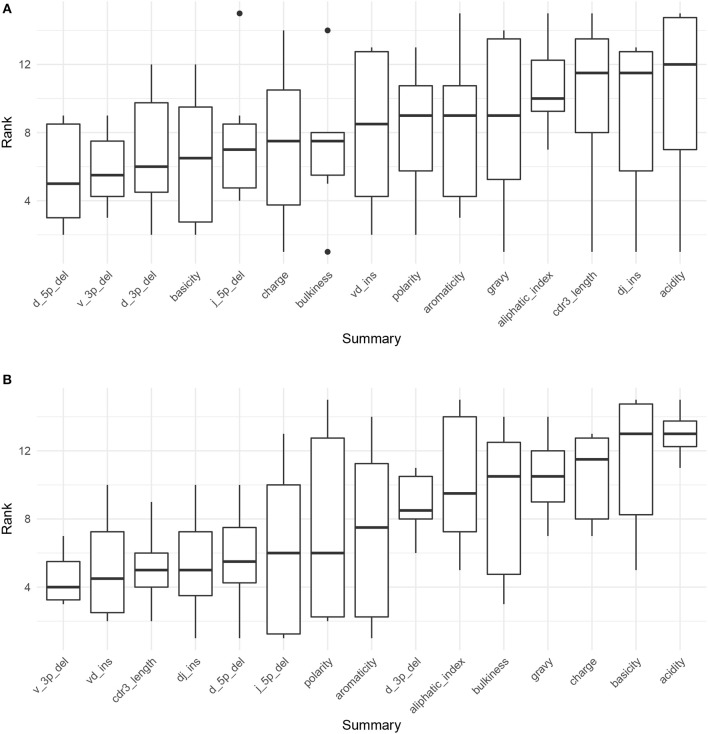
Boxplots of summary rank values taken over each dataset, in order of informativeness, as determined by the median order in which the summary branches off from the lasso paths in Figure S8, taken over each of the six paths. **(A)** Summary informativeness rank boxplots using six IGoR-annotated (34) datasets of TRB sequences. **(B)** Summary informativeness rank boxplots using six partis-annotated (18) datasets of IGH sequences.

Figure 4A displays the results of applying Algorithm 3 to IGoR annotations of TRB sequences from datasets A4_i107, A4_i194, A5_S9, A5_S10, A5_S15, and A5_S22 from Britanova et al. (34). We see that recombination-based deletion lengths comprise four of the top five summaries, with recombination-based insertion lengths, CDR3 length, and various physiochemical CDR3 properties scattered over the remaining positions. There appears to be high variability throughout the range of rankings, with the bottom three statistics all having a ranking of one for at least one coefficient vector.

Figure 4B displays the results of applying Algorithm 3 to partis annotations of IGH sequences from donors FV, GMC, and IB at timepoints −8 days and −1 h from Gupta et al. (18), downsampled to unique clonal families to avoid clonal abundance biases and decrease algorithmic runtime. We see that deletion lengths, insertion lengths, and CDR3 length comprise the top six summaries, with physiochemical CDR3 properties mostly in the bottom half of rankings. In contrast to the TCR result, there appears to be less overall variability throughout the range of rankings, with variability highest for the moderate ranking positions and notably lower for the top and bottom positions.

**Figure 5:**
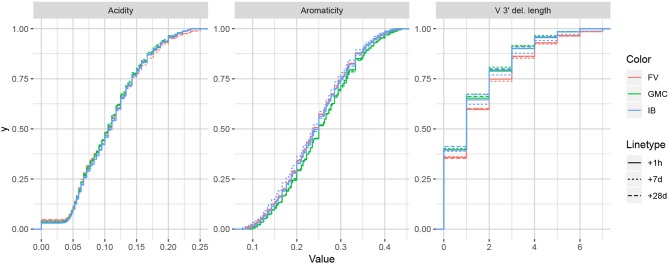
Empirical cumulative distribution functions for the bottom-, middle-, and top-ranked statistics for a validation set of partis-annotated IGH repertoires, as determined by Figure 4B.

While it is difficult to say exactly the level of correlation of each summary by the lasso result alone, since the lasso is a regularized version of least-squares, our intuition is that the nice properties of least-squares combined with the lasso's ability to eliminate less relevant coefficients leads to a subset of covariates that are generally informative. To validate this intuition, we can examine distributions of particularly ranked summaries applied to a test set of annotated repertoires not used in the model fitting. Figure 5 displays ECDFs of the acidity (bottom-ranked), aromaticity (middle-ranked), and V 3′ deletion length (top-ranked) distributions for the FV, GMC, and IB donors at timepoints +1 h, +7 days, and +28 days following an influenza vaccination (which differ from the −1 h and −8 days timepoints used for fitting), where the ranks are as determined by Figure 4B for partis-annotated IGH repertoires. Visually, we see that the acidity curves do not vary much among donors or timepoints; the aromaticity curves have slightly more variation but are still highly similar; and the V 3′ deletion length curves are more distinguished between some donors (e.g., FV and GMC) as well as some donor-timepoint interactions (e.g., +7 days and +28 days timepoints for IB). Thus, there is visual evidence that the lasso scores can identify some degree of informativeness among summaries.

### Comparing Experimental Observations to Model Simulations

sumrep can be used to validate BCR/TCR generative models, i.e., models from which one can generate (simulate) data, through the following approach. First, given a collection of AIRR-seq datasets, model parameters are inferred using the modeling software tool for each repertoire, and then these parameters are used to generate corresponding simulated datasets (Figure 1C). Next, sumrep is used to compute the summary statistics listed in Table 1 for each dataset and compare these summaries between each pair of datasets (Figure 1D). Then, a score is calculated for how well the software's simulation replicates a given summary based on how small the divergences of observed/simulated dataset pairs are compared to divergences between arbitrary observed/observed or simulated/simulated pairs.

Applying this methodology using many datasets should give a clear view of which characteristics the model captures well, as well as areas for improvement. As described in the introduction, we are motivated to do this because models are often benchmarked on simulated data, and it is important to understand discrepancies between simulated and observed data in order to properly interpret and extrapolate benchmarking results. We emphasize that validating the model in this way is different than the usual means of benchmarking model performance: rather than benchmarking the inferential results of the model, we benchmark the model's ability to generate realistic sequences.

We illustrate this approach with two case studies: an analysis of IGoR (19) applied to TRB sequences, and an analysis of partis (17, 22) simulations applied to IGH sequences. Both tools are applied to separate sets of experimental repertoires, yielding model-based annotations for each repertoire, as well as simulated datasets from the inferred model parameters for each experimental set. Summary divergences are applied to each dataset, allowing for scores for each summary to be computed for each tool.

### Assessing Summary Statistic Replication for IGoR

**Figure 6:**
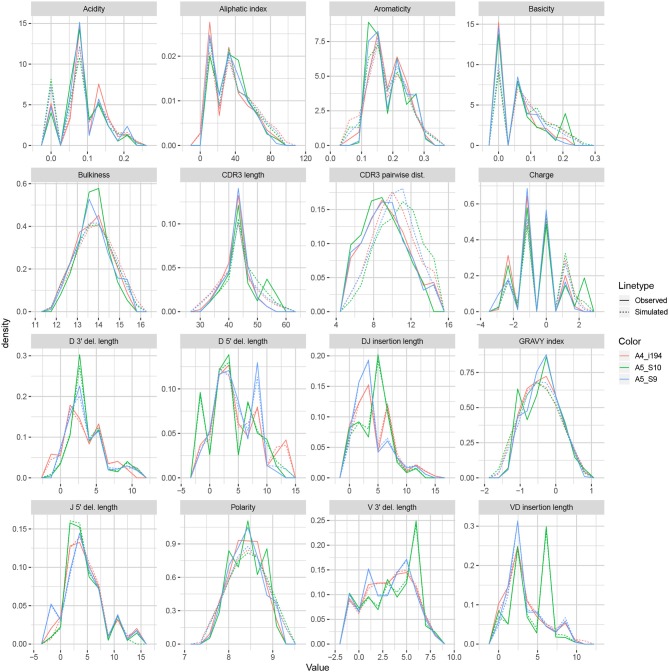
Frequency polygon plots of each univariate summary distribution for the IGoR datasets.

We apply the methodology discussed in the previous section to TRB sequences from datasets A4_i107, A4_i194, A5_S9, A5_S10, A5_S15, and A5_S22 from Britanova et al. (34). Although IGoR is typically applied to non-productive sequences in order to capture the pre-selection recombination process, for this example application we wished to understand IGoR's ability to fit the complete repertoire directly without the need for an additional selection model [e.g., (35)]. Thus, we fit the IGoR model with all sequences (which we expect to be dominated by productive sequences) and restricted the simulation to productive sequences. Figure 6 contains frequency polygons of each summary distribution for each experimental and simulated repertoire.

**Figure 7:**
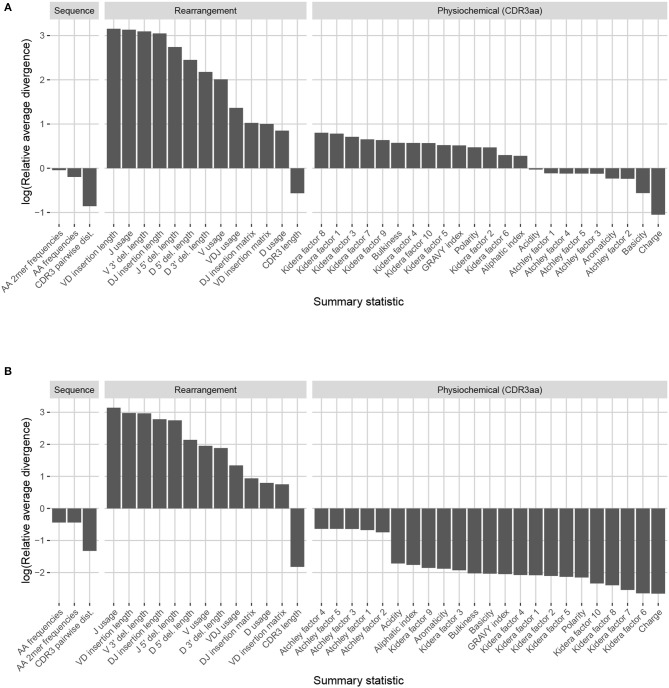
Summary scores, denoted as “log(Relative average divergence)” or “LRAD,” for each statistic in the IGoR model validation experiment. For both cases, a high score indicates a well-replicated statistic by the simulations with respect to their corresponding experimental repertoires of functional TRB sequences. **(A)** LRAD-data values for each relevant TRB statistic available from IGoR or IgBLAST as determined by Equation (8). **(B)** LRAD-sim values for each relevant TRB statistic available from IGoR or IgBLAST as determined by Equation (9).

Observation-based summary scores are computed using a log ratio of average divergences (referred to as LRAD-data, and defined in Equation 8) for a variety of TRB-relevant summaries (Figure 7A). The LRAD-data score of a summary will be high when simulations look like their corresponding observations with respect to that summary, and low when observations look more like other observations than their corresponding simulations. We exclude summaries based on sequence_alignment values (e.g., pairwise distance distributions) since IGoR does not currently have an option to output the full variable region nucleotide sequences for experimental reads.

IGoR simulations were able to recapitulate gene usage statistics of an empirical repertoire well, with J gene usage frequency being the most accurately replicated, followed by various recombination-based indel statistics. V, D, and joint VDJ gene usage are all also well-replicated, as well as both VD and DJ insertion matrices. Conversely, the CDR3 length distribution was the least accurately replicated statistic among rearrangement statistics. The Kidera factors of the CDR3 region were also replicated well, despite CDR3 length being one of the least accurately replicated statistics. Scores for other CDR3-based statistics besides Kidera factors ranged from mildly good to mildly bad, with the GRAVY index distribution being the best CDR3-based statistic (excluding Kidera factors) and charge distribution being the worst.

We also computed simulation-based summary scores (LRAD-sim, defined in Equation 9) for the same datasets and simulations (Figure 7B). The LRAD-sim score of a summary will be high when simulations look like their corresponding observations with respect to that summary, and low when simulations look more like other simulations than their corresponding observations. We still saw high scores for gene usage and indel statistics, although the CDR3 length distribution and various Kidera factor and GRAVY index distributions had much lower scores. This suggests that while the average IGoR simulation yields Kidera factor and GRAVY index distributions that look more like the observed repertoire's distributions than other observed repertoires do, these simulated repertoires still tend to produce more similar distributions to each other than to their observed counterparts. In turn, this provides an avenue of future research for TCR generative models in which certain CDR3aa properties are incorporated and expressed in simulated data.

### Assessing Summary Statistic Replication for partis

**Figure 8:**
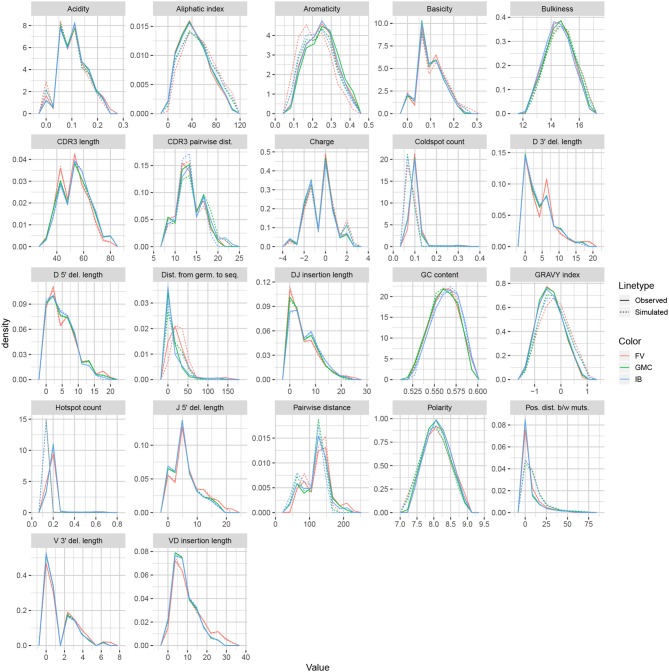
Frequency polygon plots of each univariate summary distribution for the partis datasets.

We applied the same methodology to IGH sequences from Gupta et al. (18), using datasets corresponding to the −1 h and −8 d timepoints for each of the FV, GMC, and IB donors. Figure 8 displays frequency polygons of each summary distribution for each experimental and simulated repertoire.

**Figure 9:**
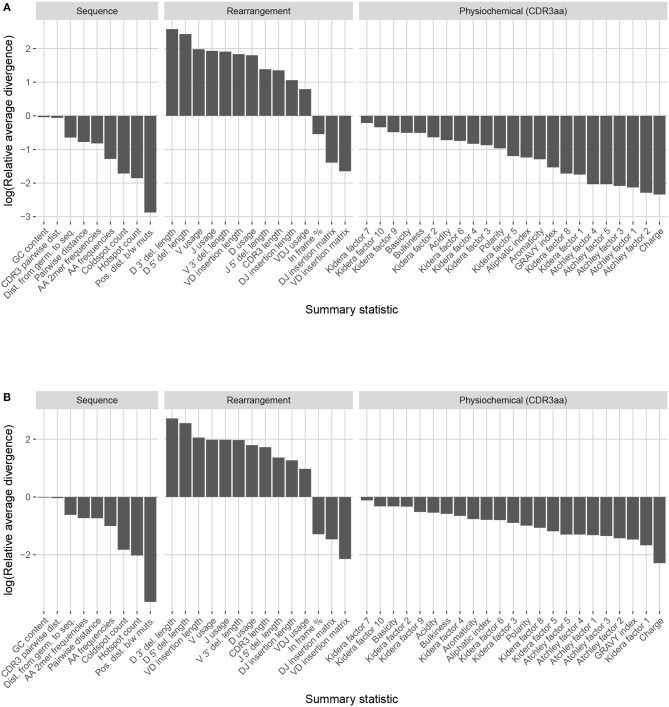
Summary scores, denoted as “log(Relative average divergence)” or “LRAD,” for each statistic in the partis model validation experiment. For both cases, a high score indicates a well-replicated statistic by the simulations with respect to their corresponding experimental repertoires of productive IGH sequences. **(A)** LRAD-data values for each relevant IGH statistic available from partis as determined by Equation (8). **(B)** LRAD-sim values for each relevant IGH statistic available from partis as determined by Equation (9).

Observation-based summary scores were computed using the LRAD-data Equation (8) for a variety of IGH relevant summaries (Figure 9A).

Like IGoR, we see that partis simulations also excelled at replicating gene usage and recombination statistics, while additionally replicating CDR3 length distributions well. However, partis struggled to recapitulate VD and DJ insertion matrices, which it does not explicitly include in its model. This contrasts with IGoR which incorporates these insertion matrices during model fitting, and thus recapitulates these matrices well. The other statistics yielded scores ranging from slightly to very negative, with many mutation-based summaries like positional distance between mutations and hot and cold spot counts being poorly captured. The low scores of mutation-based summaries may arise from the decision to select a single representative from each clonal family, which itself arises from the complications in matching clonal family abundance distributions of simulations to data. This makes it difficult to identify the exact contributions of these factors to the summary discrepancies. Nonetheless, this suggests that these sorts of quantities may need to be more explicitly accounted for in BCR generative models if more realistic simulations are desired.

We also computed simulation-based summary scores (LRAD-sim, defined in Equation 9) for the same datasets and simulations (Figure 9B). The scores are highly similar to those seen in Figure 9A, with some summaries seeing a moderate drop.

## Methods

### Divergence

We use the Jenson-Shannon (JS) divergence for comparing distributions of scalar quantities, which constitutes most summaries in sumrep. The Jenson-Shannon divergence of probability distributions *P* and *Q* with densities *p*(·) and *q*(·) is a symmetrized Kullbeck-Leiber divergence, defined as

(1) $$\begin{array}{r} \text{JSD}\left(P\;||\;Q\right):=\frac{\text{KLD}\left(P||M\right)+\text{KLD}\left(Q||M\right)}{2} \end{array}$$

where *M*: = (*P* + *Q*)/2 and KLD(*P*||*M*) is the usual KL-divergence,

(2) $$\begin{array}{r} \text{KLD}\left(P_{1}\;||\;P_{2}\right):=E_{\text{X}\sim P_{1}}\left[\log\left(\frac{p_{1}\left(\text{X}\right)}{p_{2}\left(\text{X}\right)}\right)\right]. \end{array}$$

In the case where *P* and *Q* are both discrete distributions, this becomes

(3) $$\begin{array}{r} \text{KLD}\left(P_{1}\;||\;P_{2}\right)=\underset{i\in \text{supp}\left(P_{1}\right)}{\sum }p_{1}\left(i\right)\log\left(\frac{p_{1}\left(i\right)}{p_{2}\left(i\right)}\right) \end{array}$$

where supp(*P*) is the countable support of distribution *P*. Because the discrete formulation has computational benefits over the continuous one, we discretize continuous samples and treat them as discrete data. By default, we use $B=\max\left(\sqrt{\min\left(m,n\right)},2\right)$ bins of equal length, where *m* = |supp(*P*)| and *n* = |supp(*Q*)|, which is designed to scale with the complexity of *m* and *n* simultaneously. We also discard bins which would lead to an infinite KL divergence for numerical stability.

For counts of categorical data, we instead appeal to the sum of absolute differences, or ℓ_1_ divergence, for comparison:

(4) $$\begin{array}{r} d_{\ell_{1}}\left(R_{1},R_{2};c,S\right)=\underset{s\in S}{\sum }|c\left(s;R_{1}\right)-c\left(s;R_{2}\right)|. \end{array}$$

In words, Equation (4) iterates over each element *s* in some set S, calculates the count *c* of *s* within repertoires *R*_1_ and *R*_2_, respectively, takes the absolute difference of counts, and appends this to a rolling sum. This metric is well suited for comparing marginal or joint V/D/J-gene usage distributions. For example, if V, D, and J represent the germline sets of V, D, and J genes, respectively, define usage *u* of gene triple (v,d,j)∈V×D×J for repertoire *R* as

(5) $$\begin{array}{r} u\left(R;v,d,j\right)=\#\left\{s\in R:s_{v}=v,s_{d}=d,s_{j}=j\right\}, \end{array}$$

where e.g., *s*_*v*_ = the V gene of *s*. Then an appropriate divergence for the joint VDJ gene usage for repertoires *R*_1_ and *R*_2_ is

(6) $$\begin{array}{r} d\left(R_{1},R_{2};u,V,D,J\right)=\underset{v\in V}{\sum }\underset{d\in D}{\sum }\underset{j\in J}{\sum }|u\left(v,d,j;R_{1}\right)-u\left(v,d,j;R_{2}\right)|. \end{array}$$

The ℓ_1_ divergence is also relevant for computing amino acid frequency and 2mer frequency distributions. Note that we can normalize the counts to become relative frequencies and apply (4) on the resultant scale which may be better suited to the application, especially when dataset sizes differ notably.

### Approximating Distributions via Subsampling and Averaging

Computing full summary distributions over large datasets can be intractable. However, we can compute a Monte Carlo distribution estimate by repeatedly subsampling and aggregating summary values until convergence. Algorithm 1 formalizes this idea, appending batch samples of the full distribution *d* to a rolling approximate distribution and terminating when successive distribution iterates have a JS divergence smaller than tolerance ε. Note that continually appending values to a rolling vector is analogous to computing a rolling average, where the subject of the averaging is an empirical distribution rather than a scalar.

**Algorithm 1:** Compute automatic approximate distribution

**Input:** repertoire *R*, summary *s*, batch size *m*, convergence tolerance ε
**Output:** subsampled approximation to the full distribution *d* of *R*
*R*_0_ ← subsample(*R, m*)
*d*_0_ ← *s*(*R*_0_)
*n* ← 1
error ← ∞
**while** error > ε **do**:
*R*_samp_ ← subsample(*R, m*)
*d*_samp_ ← *s*(*R*_samp_)
*d*_*n*_ ← concatenate(*d*_*n*−1_, *d*_samp_)
error ← JSD(*d*_*n*−1_, *d*_*n*_)
*n* ← *n* + 1
**return** *d*_*n*_

An alternative would be to simply compute the distribution on one subsample of the data and use this as a proxy distribution. The main advantage of Algorithm 1 over such an approach is that it provides a sense of convergence to the full distribution via the tuning parameter ε, while automatically determining the size of the necessary subsample. The algorithm can also be tuned according to batch size *m*, which sumrep takes to be 30 by default. We conduct a performance analysis of Algorithm 1 in Appendix A and empirically demonstrate efficiency gains in a variety of realistic settings without sacrificing much accuracy.

Some summaries induce distributions for which Algorithm 1 is inherently ill-suited. This occurs when a summary applied to a subset of a dataset does not follow the same distribution as the summary applied to the full dataset. For example, consider the nearest neighbor distance of a sequence *s*_*i*_ with respect to a multiset of sequences *R* (i.e., elements in *R* can have multiplicity ≥ 1),

(7) $$\begin{array}{r} d_{\text{NN}}\left(s_{i},R\right):=\underset{s\in R\backslash \left\{s_{i}\right\}}{\min}d\left(s_{i},s\right), \end{array}$$

where *d*(·, ·) is a string metric (e.g., the Levenshtein distance). If we take any subset *S* of *R*, then *d*_NN_(*s*_*i*_, *S*) ≥ *d*_NN_(*s*_*i*_, *R*) ∀*i*, since *R* will have the same sequences to iterate over, and possibly more sequences, which can only result in the same or a smaller minimum.

In this case, we can still obtain an unbiased approximate to the nearest neighbor distance distribution using the following modification of Algorithm 1. For each iteration, sample a small batch *B* = (*s*_1_, …, *s*_*b*_) of *b* sequences, and compute *d*_NN_ of each *s*_*i*_ to the full repertoire *R*. Since each batch *B* computes the exact nearest neighbor with respect to *R*, we get the true value of *d*_NN_ for each *s*∈*B*. The gain in efficiency stems from the fact that we only compute this true *d*_NN_ for a subsample of the sequences of the full repertoire *R*. Thus, appending batches to a running distribution until convergence as in Algorithm 1 will produce increasingly refined, unbiased approximations as the tolerance decreases. Algorithm 2 explicates this procedure.

**Algorithm 2:** Compute automatic approximate nearest neighbor distance distribution

**Input:** repertoire *R*, distance *d*, batch size *m*, convergence tolerance ε
**Output:** subsampled approximation to the full nearest neighbor distribution *d*_NN_ of *R*
*d*_0_ ← DOBATCHSTEP(*R, m*)
*n* ← 1
error ← ∞
**while** error > ε **do**:
*d*_samp_ ← DOBATCHSTEP(*R, m*)
*d*_*n*_ ← concatenate(*d*_*n*−1_, *d*_samp_)
error ← JSD(*d*_*n*−1_, *d*_*n*_)
*n* ← *n* + 1
**return** *d*_*n*_
**function** DOBATCHSTEP(*R, m*)
**for** *i* = 1, …, *m* **do**:
*s*_*i*_ ← subsample(*R*, 1)
*d*_*i*_ ← *d*_NN_(*s*_*i*_; *R*)
**return** (*d*_1_, …, *d_m_*)

Algorithm 2 may yield a high runtime if *R* is large, the sequences in *R* are long, or the tolerance ε is small. Nonetheless, we empirically demonstrate in Appendix B that in the case of typical BCR sequence reads, even very small tolerances incur reasonable runtimes, and when *R* is large, the algorithm is orders of magnitude faster than computing the full distribution over *R*.

We show that the efficiency and accuracy of these algorithms vary by summary statistic in Appendix B, and identify appropriate defaults accordingly. Specifically, sumrep uses ε = 0.001 for arbitrary summary approximation routines and ε = 10^−4^ for getNearestNeighborDistribution. Moreover, sumrep retrieves approximate distributions by default only for getPairwiseDistanceDistribution, getNearestNeighborDistribution, and getCDR3PairwiseDistanceDistribution.

### Summary Statistic Informativeness Ranking

To quantify the relative informativeness of various summary statistics in distinguishing between different datasets, we perform a multinomial lasso regression where covariates are sequence-level summaries and the response is dataset identity. Since ℓ_1_ multinomial regression outputs a separate coefficient vector **β** for each response value, we aggregate by taking medians of each dataset-specific lasso ordering for each summary to get the final score. This also yields a range of rankings to assess the variation in scores by summary and by inferential model (e.g., partis, IGoR). In the case of ties, we randomize rankings to avoid alphabetization biases or other similar artifacts. Detailed pseudocode is provided in Algorithm 3.

**Algorithm 3:** Rank summary statistics by informativeness

**Input:** annotations datasets *d*_1_, …, *d*_*D*_, sequence-level summaries **s**(·) = [*s*_1_(·), …, *s*_*S*_(·)], lasso parameters λ_1_, …, λ_Λ_
**Output:** A vector of ranks for the summaries
**for** *d* = *d*_1_, …, *d*_*D*_ **do:**
**X**_*d*_ ← [**s**(*d*_1_), …, **s**(*d*_*D*_)]
$X\;\leftarrow \;\left[\begin{array}{l} X_{d_{1}}\\ \;\vdots \\ X_{d_{D}} \end{array}\right]$
$y\leftarrow \left[\begin{array}{c} \text{rep}{\left(1,\text{rows}(d_{1})\right)}^{T}\\ \vdots \\ \text{rep}{\left(D,\text{rows}(d_{D})\right)}^{T} \end{array}\right]$
⊳ rows(*d*_*i*_) is the number of sequences in the *i*th dataset
**for** λ = λ_1_, …, λ_Λ_ **do:**
$\left(\beta_{d_{1}}^{\lambda },\ldots ,\beta_{d_{D}}^{\lambda }\right)\leftarrow \text{MultinomialLasso}\left(\text{X},\text{y};\lambda \right)$
**for** *d* = *d*_1_, …, *d_D_* **do:**
**for** *s* = *s*_1_, …, *s*_*S*_ **do:**
*t*_*d, s*_ ←
$\min\left(\min\left\{\lambda_{1}\leq \lambda \leq \lambda_{\Lambda }:\beta_{d,s}^{\lambda }>0\forall t>\lambda \right\},\infty \right)$
**r***_d_* = rank(*t_d_*, _s1_, …, *t_d, sS_*)
**R** = (**r***d*_1, …,_ **r**_*d_D_*_)
scores = rank(median_*s*_1__(**R**), …, median_*s_S_*_(**R**))
**return** scores

This approach only works for sequence-level summaries *s* ∈ ℝ^*n*^ for a dataset *d* of *n* = rows(*d*) sequences in order to form a well-defined design matrix $\text{X}\in {\mathbb{R}}^{\left(\overset{D}{\underset{i=1}{\sum }}\text{rows}\left(d_{i}\right)\right)\times S}$ over all datasets *d* = *d*_1_, …, *d*_*D*_ under consideration. For example, it is unclear how to incorporate the pairwise distance distribution, which is not a sequence-level summary, as a covariate, since this summary in general yields a column of a larger length than the number of sequences. Still, as most summaries considered above can be applied at the sequence level, this method greatly reduces the number of summaries the user needs to examine.

### Model Validation of IGoR

We used the -infer subcommand of IGoR to fit custom, dataset-specific models for each experimental dataset. Since we were interested in many CDR3-based statistics and IGoR does not currently include inferred CDR3 sequences with rearrangement scenarios, we used IgBLAST to extract CDR3s for each sequence. For each sequence, we considered only the rearrangement scenario with the highest likelihood as determined by IGoR. When a list of more than one potential genes was given as the gene call, we considered only the first gene in the list. Several fields were renamed to match the AIRR specification when the definitions align without ambiguity. As described in Results, we trained on productive sequences and restricted the simulation to productive sequences.

We applied IGoR in this way to six datasets of TRB sequences from Britanova et al. (34), which studied T cell repertoires from donors ranging from newborn children to centenarians.

### Model Validation of partis

We used partis to infer custom generative models for each experimental dataset. We ran the partition subcommand to incorporate underlying clonal family clustering among sequences during inference, and then downsampled each observed and simulated dataset so that each clonal family is represented by one sequence. Since partis returns a list of the top most likely annotations scenarios for each rearrangement event, we considered only the scenario with the highest model likelihood for each sequence. We denote the indel_reversed_seqs field as sequence_alignment and naive_seq as germline_alignment as they satisfy these definitions from the AIRR Rearrangement schema. Several other fields are renamed to match the AIRR specification when the definitions align without ambiguity.

Before running summary comparisons, we randomly downsample to one receptor per clonal family to get a dataset consisting of unique clonotypes for both the observed and simulated datasets. We do this since partis simulate draws from distributions over clonal families for each rearrangement event as inferred from partis partition. While it is possible to simulate multiple leaves for each rearrangement, it is not obvious how to best synchronize this with the observed clonal family distributions. A more involved analysis would attempt to mimic the clone size distribution in data as closely as possible, potentially with correlations between clone size and other rearrangement parameters, and assess sequence-level statistics within each clonal family. Here we opt to subsample to unique clones and avoid abundance biases altogether.

We applied partis in this way to six datasets of IgH sequences from Gupta et al. (18), which studied B cell repertoires from donors prior to and following an influenza vaccination.

### Scoring Summary Statistic Replication by Model

We wish to measure how well a given statistic is replicated when a model performs simulations using parameters inferred from an observed repertoire dataset. One approach is to score the statistic *s* based on the average divergence of observations to their simulated counterparts when applying *s*(·), and the average divergence of observations to other observations when applying *s*(·). Suppose we have *k* experimental repertoires of immune receptor sequences, and let *R*_*i*, obs_ and *R*_*i*, sim_, 1 ≤ *i* ≤ *k*, denote the *i*th observed and simulated repertoire, respectively. For a given statistic *s*, let $D_{s}\left(R_{1},R_{2}\right)$ be the divergence of repertoires *R*_1_ and *R*_2_ with respect to *s*. We can score a simulator's ability to recapitulate *s* from the observed repertoire to the simulated via the following log relative average divergence (LRAD):

(8) $$\begin{array}{r} \text{LRAD-data}\left(s\right):\;=\log\left(\frac{\frac{1}{\frac{1}{2}k\left(k-1\right)}\sum_{i=1}^{k}\sum_{j\neq i}D_{s}\left(R_{i,\text{obs}},R_{j,\text{obs}}\right)}{\frac{1}{k}\sum_{i=1}^{k}D_{s}\left(R_{i,\text{obs}},R_{i,\text{sim}}\right)}\right). \end{array}$$

For a given summary *s*, LRAD-data will be positive if the simulated repertoires tend to look more like their experimental counterparts in terms of this summary than experimental repertoires look like other experimental repertoires, and negative if experimental repertoires tend to look more like other experimental repertoires than they do their simulated counterparts. In other words, LRAD-data scores how well a simulator can differentiate *s* from an experimental repertoire among other repertoires, and recapitulate *s* into its simulation. Applying the log to the ratio allows for the magnitudes of scores to be directly comparable (so that a summary with score *a*> 0 performs as well as a summary with score −*a* < 0 performs poorly).

Another related score compares the average divergence of observations to their simulated counterparts, and the average divergence of simulations to other simulations. Formally, this becomes

(9) $$\begin{array}{r} \text{LRAD-sim}\left(s\right):\;=\log\left(\frac{\frac{1}{\frac{1}{2}k\left(k-1\right)}\sum_{i=1}^{k}\sum_{j\neq i}D_{s}\left(R_{i,\text{sim}},R_{j,\text{sim}}\right)}{\frac{1}{k}\sum_{i=1}^{k}D_{s}\left(R_{i,\text{obs}},R_{i,\text{sim}}\right)}\right) \end{array}$$

where the difference from (8) is that the divergences in the numerator are applied to simulated-simulated dataset pairs rather than observed-observed dataset pairs. LRAD-sim for a given summary will be positive if simulated repertoires tend to look more like their experimental counterparts in terms of this summary than simulated repertoires look like other simulated repertoires, and negative if the simulated repertoires tend to look more alike.

These scores underlie the model validation analyses of partis and IGoR simulations in the Results section, and comprise the values displayed in Figures 7, 9. However, this framework can be used to validate any immune receptor repertoire simulator which outputs the fields compatible with the summaries in Table 1, or more generally any set of summaries generated by a model-based simulator that is not supported directly by sumrep.

A feature of our methodology is that we use the same tool to produce simulations that we used to produce the annotations. To examine the sensitivity of this method, we performed a separate analysis by obtaining dataset annotations from standalone IgBLAST (36), and comparing these to simulations based on partis annotations using IMGT germline databases. This is discussed in detail in Appendix E; in particular, we find that scores differ to varying extents between the tools, and argue that while there are probably some biases when using a common tool for annotations and simulations, this is also driven by the differences in the nature of the tools' specifications. We did not perform a similar analysis for IGoR annotations since IgBLAST was used to infer CDR3s within the IGoR workflow.

### Materials

The raw data for the TCR summary divergence MDS analysis comes from Pogorelyy et al. (32), which was postprocessed into a suitable format for analysis. For each donor-timepoint combination, a single blood draw was split in replicas at the level of cell mixture.

The raw data for the BCR summary divergence MDS analysis comes from Rubelt et al. (33); IgBLAST-preprocessed data was downloaded from VDJServer in the AIRR format. For quality control, sequences with a run of 3 or more N bases in the raw sequence were discarded.

For the TCR model validation analysis, we use six datasets from Britanova et al. (34), corresponding to labels A4_i107, A4_i194, A5_S9, A5_S10, A5_S15, and A5_S22. For tractability purposes, we chose the six datasets with the fewest number of sequence reads; the number of reads from these six datasets used in the analysis ranged from 37,363 sequences to 243,903 sequences. These datasets consist of consensus RNA sequences assembled using UMIs. Most of these sequences are productive; as previously described, for this example application we are benchmarking IGoR's ability to fit complete repertoires rather than only non-productive repertoires.

The data for the BCR model validation analyses originated from samples first sequenced and published in Laserson et al. (37), although we used the Illumina MiSeq data published in Gupta et al. (18) for our analyses. These datasets represent repertoires of three human donors from multiple time points following an influenza vaccination. We use datasets from time points −1h and −8d for the FV, GMC, and IB donors for the summary informativeness and partis model validation analyses; the +1h, +7d, and +28d datasets for the FV, GMC, and IB donors for the summary informativeness validation; and the FV -1 h dataset for the approximation routine performance analyses in Appendices A, B.

## Conclusions

We have presented a general framework for efficiently summarizing, comparing, and visualizing AIRR-seq datasets, and applied it to several questions of scientific interest. One can imagine many further applications of sumrep, as well as promising avenues of research: contrasting repertoires in the context of antigen response or vaccination design and evaluation may shed some light on which summaries can distinguish between such covariates; and comparing the summary distributions of naive repertoires from multiple healthy individuals is likely to aid our understanding of the patterns of variability exhibited by “normal” repertoires, which in turn may aid the detection of repertoire abnormalities. sumrep could also be used to evaluate the extent to which artificial lymphocyte repertoires look like natural ones (38).

There are several other packages dedicated to detailed summaries and visualization of immune receptor repertoires. The tcR (39) and bcRep (40) packages for R include methods for retrieving and comparing gene usage summaries, computing clonotype diversity indices, and visualizing various repertoire summaries. VDJtools (41) is a command line tool which performs similar repertoire summarization, comparison, and visualization tasks for TCR data. Desktop GUI-based programs include ImmunExplorer (42) and Vidjil (43). Vidjil is also available as a webserver, as is ASAP (44). Antigen Receptor Galaxy (45) offers online access to many analysis tools. These tools have a subset of the summary statistics described here, and do not have the comparative analysis features of sumrep. The IGoR (19) software features an algorithm for summarizing statistics of the V(D)J rearrangement process; however, its main focus is on learning the basic model for non-productive T- and B-cell repertoire and it does not provide any built-in methods for comparing inferred models between datasets.

A natural extension of the model validation in this report would be to assess the performance of many competing repertoire analysis tools over a larger group of datasets. sumrep can be also used to detect systemic biases between different library preparation protocols and control for batch effects that can confound meta-analysis of AIRR-Seq data. Moreover, while many of the summaries are applied to the CDR3 region by default, it would be interesting to perform separate analyses restricted to different CDRs and framework regions, as physiochemical characteristics of these regions can differ greatly.

Finally, although sumrep already supports the AIRR rearrangement schema by default, we plan to thoroughly integrate sumrep as a downstream analysis tool for any AIRR-compliant software or workflow.

## Data Availability Statement

Publicly available datasets were analyzed in this study. This data can be found here: PRJNA316572, PRJNA349143, PRJNA493983, SRP065626.

## Author Contributions

BO, PM, CS, DR, JV, MS, AS, WL, and FM conceived of the project and guided the overall design of the software and analyses. BO designed and implemented the main software package. BO, PM, CS, and AO performed computational analyses. BO and FM wrote the first draft of the manuscript. All authors contributed to manuscript revision, read, and approved the submitted version.

### Conflict of Interest

JV is employed by Genentech Inc. The remaining authors declare that the research was conducted in the absence of any commercial or financial relationships that could be construed as a potential conflict of interest. The reviewer SC and handling editor declared their shared affiliation.
