# Supplementary material for: sumrep: A Summary Statistic Framework for Immune Receptor Repertoire Comparison and Model Validation
Source: Front Immunol. 2019 Nov 1;10:2533. doi: 10.3389/fimmu.2019.02533 (PMC6838214; doi:10.3389/fimmu.2019.02533)
Supplement: Supplementary file 1 [file Data_Sheet_1.pdf]

Appendices to "`sumrep`: a summary statistic framework for immune receptor repertoire comparison and model validation"

Appendix A: Performance analysis of Algorithm 1

Here, we run Algorithm 1 on the `partis`-annotated FV -1h dataset (henceforth referred to as `p_f1`), subsampled without replacement to 10,000 sequences for tractability. We compute the pairwise distance distribution of CDR3 sequences for the full subsampled dataset, and approximate distributions with tolerances $\varepsilon \in \{0.1, 0.001, \ldots, 10^{-7}\}$. We replicate this experiment for 10 trials so that the subsampled dataset remains the same, but a new instance of the subsampling algorithm is run each time. Figure S1a shows a frequency polygon of each distribution and figure S1b shows their empirical cumulative distribution functions. We see that the approximate distributions appear to converge to the full distribution as the tolerance gets smaller. Figure S1c displays the KL-divergence to the true distribution for each tolerance, again indicating convergence to the truth. Figure S1d displays the runtimes and log-runtimes for each tolerance as well as the true "population" runtime for the full dataset; while the runtime grows exponentially as $\varepsilon \to 0$, the approximation algorithm is still much faster than computing the full distribution for each considered value of ε.

Next we investigate the effect of dataset size on the performance of Algorithm 1. For sample sizes $n \in \{\exp(6), \ldots, \exp(10)\}$, we subsample `p_f1` without replacement to n sequences and compute the pairwise distance distribution of CDR3 sequences for the full subsampled dataset as well as those given by tolerances $\varepsilon \in \{0.1, 0.01, ..., 10^{-5}\}$. We perform this experiment 10 times for each n. Boxplots of the KL-divergence by $\log(n)$ and tolerance over all trials are displayed in Figure S2a. We see no obvious trend in the effect of dataset size on the KL-divergence for any choice of tolerance for the pairwise distribution. Boxplots of the runtime (in log-seconds) by log(size) and tolerance are shown in Figure S2b, showing that runtime increases with sample size for high tolerance, but tends towards a constant runtime by sample size as tolerance decreases. Boxplots of the log-efficiency by log(size) and tolerance are shown in Figure S2c, where

$$\text{Efficiency} := \frac{\text{time to compute full distribution}}{\text{time to compute approximate distribution}}. \tag{1}$$

Here we plot efficiency on a log scale, so that the line $y = 0$ corresponds to instances when the true and approximate routines have identical runtimes. Thus, the region $y > 0$ corresponds to instances when Algorithm 1 outperforms the computation of the full nearest neighbor distribution. For moderate to large datasets and reasonable choices of ε, the approximate routine is much more efficient than computing the full distribution. Efficiency also appears to increase exponentially with dataset size, although decreases at least exponentially as tolerance decreases. Nonetheless, the accuracy of Algorithm 1 applied to the pairwise distance distribution is scalable to large datasets while leading to large gains in runtime efficiency for reasonable choices of ε.

Finally, we investigate the effect of summary statistic on the performance of Algorithm 1. We run the algorithm for the pairwise distance, GC content, hotspot count, coldspot count, and distance from germline to sequence distributions on `p_f1` subsampled without replacement to 10,000 rows. For each summary, we run the algorithm for tolerances $\varepsilon \in \{0.1, \ldots, 10^{-5}\}$. We perform this experiment 10 times for each (summary, ε) combination. Figures S3a, S3b, and S3c show the KL-divergence to the full dataset distributions, runtimes, and efficiencies, respectively, by summary and tolerance over all trials. We see that the KL divergence, runtime, and efficiency of the approximation routine depends on the summary in question. In particular, the approximation routine for hotspot and coldspot count distributions does not yield as high of an efficiency for moderately low tolerance, and struggles to minimize the KL-divergence to the true distribution for higher

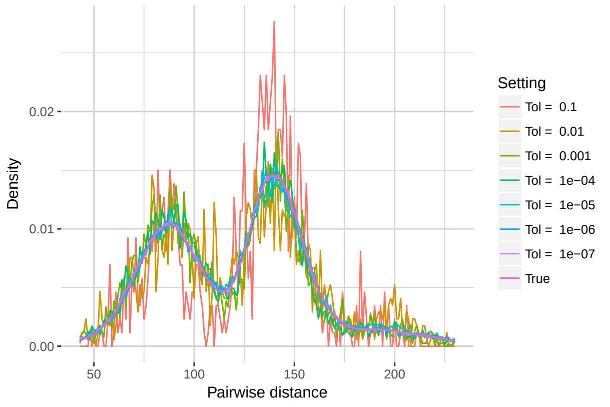

(a) Frequency polygons of true and subsampled pairwise distance distributions by tolerance.

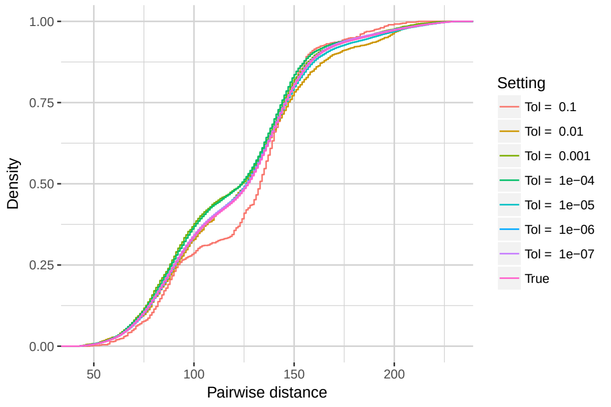

(b) ECDF of true and subsampled pairwise distance distributions by tolerance.

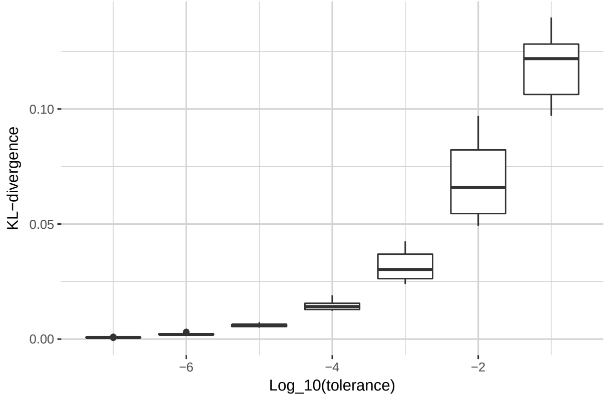

(c) KL-divergence to true pairwise distance distribution by tolerance, taken over 10 trials of the algorithm.

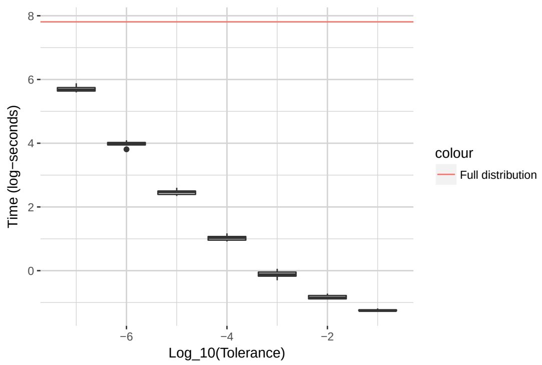

(d) Runtime (in log-seconds) for Algorithm 1 by tolerance, taken over 10 trials.

Figure S1: Performance of Algorithm 1 by tolerance applied to the pairwise distance distribution.

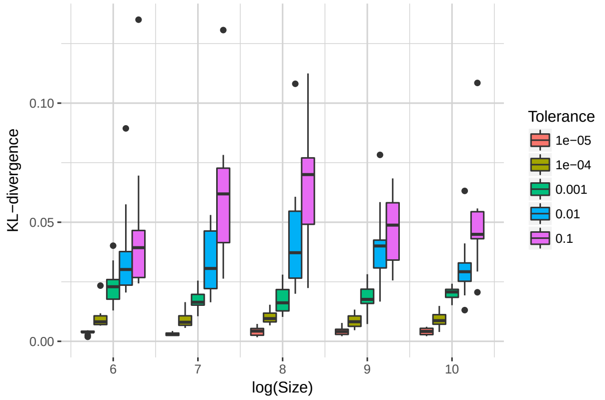

(a) KL-divergence to true pairwise distance distribution by tolerance and log(size) of dataset, taken over 10 trials of the algorithm.

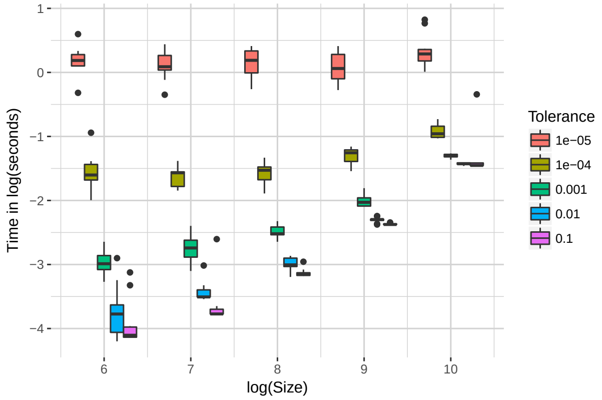

(b) Runtime by tolerance and log(size) of dataset, taken over 10 trials of the algorithm.

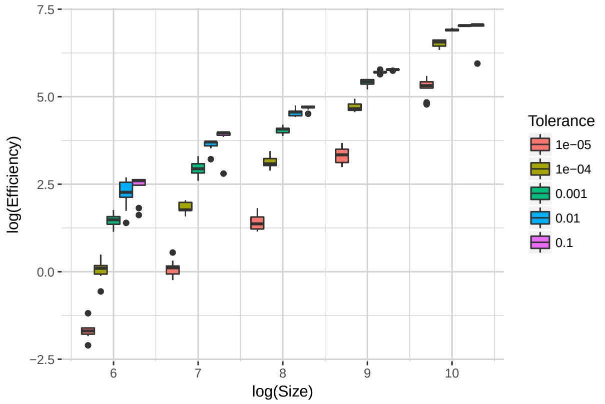

(c) Efficiency by tolerance and log(size) of dataset, taken over 10 trials of the algorithm.

Figure S2: Performance of Algorithm 1 by sample size and tolerance applied to the pairwise distance distribution.

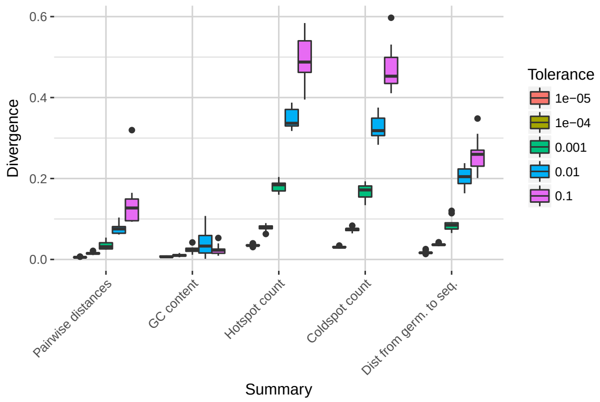

(a) KL-divergence to true summary distributions by tolerance, taken over 10 trials of the algorithm

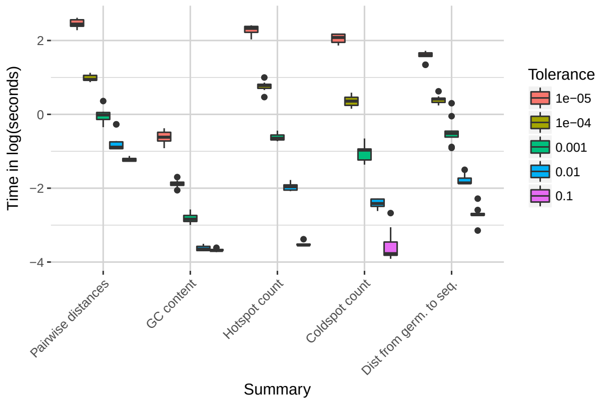

(b) Runtime by summary distribution and tolerance, taken over 10 trials of the algorithm

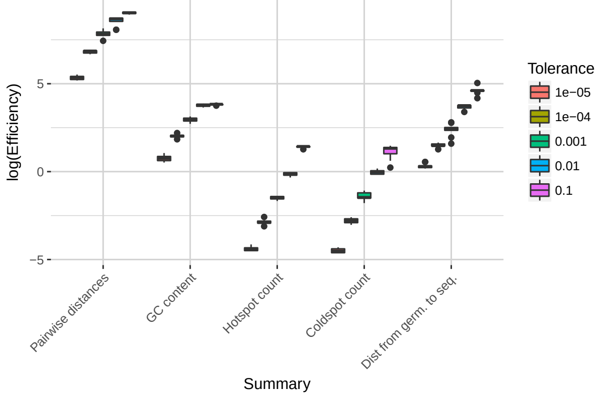

(c) Efficiency by summary distribution and tolerance, taken over 10 trials of the algorithm

Figure S3: Performance of Algorithm 1 by summary statistic and tolerance applied to the pairwise distance distribution.

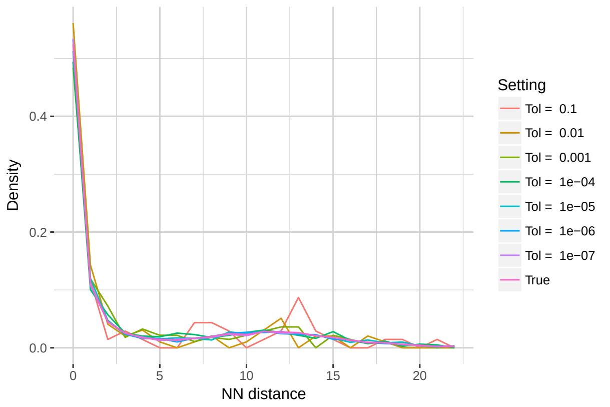

(a) Frequency polygons of true and subsampled nearest neighbor distance distributions by tolerance.

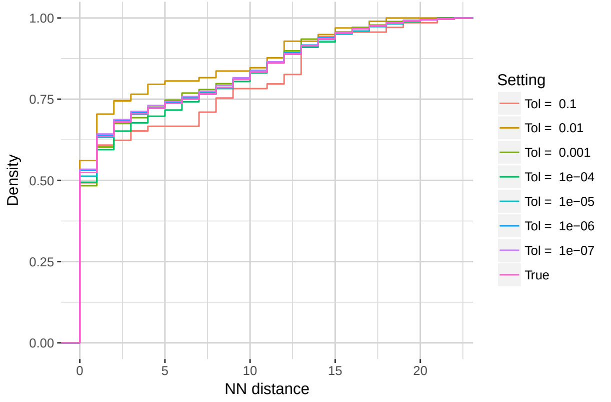

(b) ECDF of true and subsampled nearest neighbor distance distributions by tolerance.

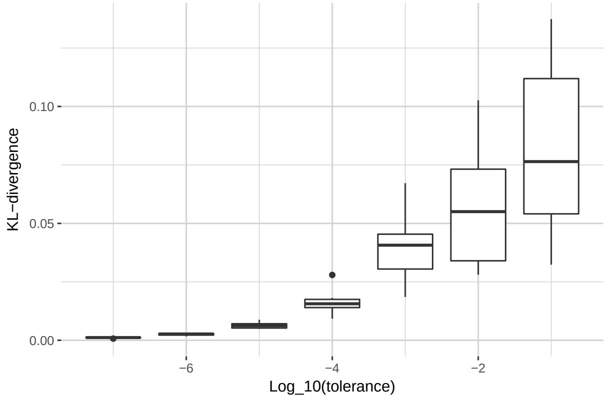

(c) KL-divergence to true nearest neighbor distance distribution by tolerance, taken over 10 trials of the algorithm.

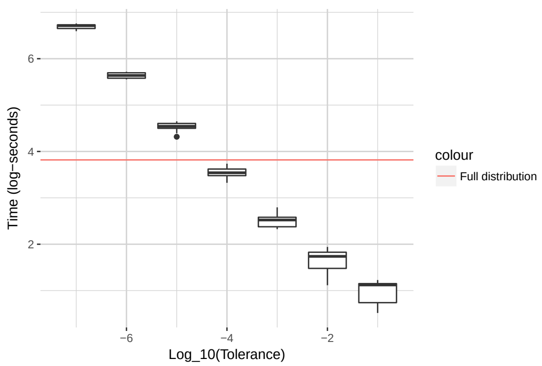

(d) Runtime (in seconds) and log-runtime (in log-seconds) for Algorithm 2 by tolerance, taken over 10 trials.

Figure S4: Performance of Algorithm 2 by tolerance applied to the nearest neighbor distribution of CDR3nt sequences.

tolerances. This is likely due to the fact that the full hotspot and coldspot count distributions is extremely fast to compute even for large datasets.

These results suggest that convergence and efficiency will vary by summary, and the user should be aware of this fact when choosing whether to run the approximation routine as well as an appropriate tolerance. By default, `sumrep` uses $\varepsilon = 0.001$ for arbitrary summary approximation routines, and retrieves approximate distributions by default only for `getPairwiseDistanceDistribution`, `getNearestNeighborDistribution`, and `getCDR3PairwiseDistanceDistribution`.

Appendix B: Performance analysis of Algorithm 2

Here, we assess the modification of the distribution approximation routine for the nearest neighbor distribution. We run Algorithm 2 on `p_f1` subsampled without replacement to 10,000 sequences for tractability. We compute the nearest neighbor distribution of CDR3 nt sequences for the full subsampled dataset, and approximate distributions with tolerances $\varepsilon \in \{0.1, 0.001, \dots, 10^{-7}\}$. We replicate this experiment for 10 trials in the same manner as detailed in Appendix A.

Figure S4a shows a frequency polygon of each distribution, and Figure S4b shows their empirical cumulative distribution functions. Figure S4c shows KL divergences of approximate distributions to the true distribution which decay as $\varepsilon \to 0$. Indeed, these three figures indicate that the approximate distributions converge to the full distribution as $\varepsilon \to 0$. Figure S4d displays boxplots of the runtime in log-seconds for

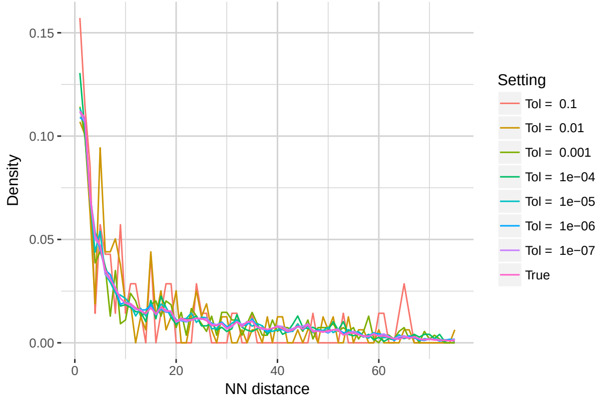

(a) Frequency polygons of true and subsampled nearest neighbor distance distributions by tolerance.

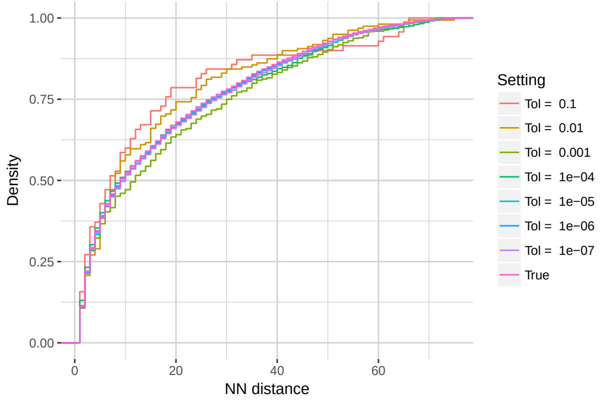

(b) ECDF of true and subsampled nearest neighbor distance distributions by tolerance.

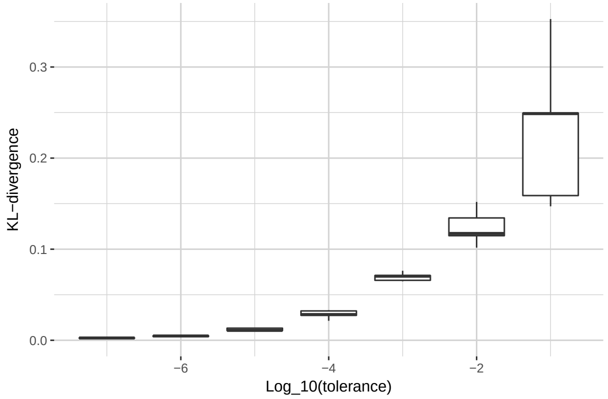

(c) KL-divergence to true nearest neighbor distance distribution by tolerance, taken over 10 trials of the algorithm.

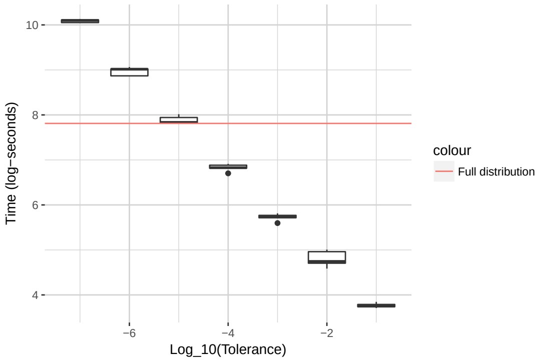

(d) Runtime (in seconds) and log-runtime (in log-seconds) for Algorithm 1 by tolerance, taken over 10 trials.

Figure S5: Performance of Algorithm 2 by tolerance applied to the nearest neighbor distribution of pairwise-aligned VDJ sequences.

Algorithm 2 as well as the runtime to compute the full distribution. In this case, we see that the Algorithm 2 becomes slower than computing the full distribution when $\varepsilon \lesssim 10^{-5}$.

To assess the effect of sequence lengths on Algorithm 2, we perform the same experiment as above on pairwise aligned VDJ sequences (via the `sequence_alignment` column rather than inferred CDR3 sequences. These length distributions are different by about an order of magnitude. We note that the pairwise aligned VDJ sequences are the default for Algorithm 2 within `sumrep`, although we anticipate users to examine this distribution for CDR3s as well as full V(D)J sequences. We run Algorithm 2 on the same subsampled 10,000 sequences of `p_f1`.

Figure S5a shows a frequency polygon of the same distributions, and Figure S5b shows their empirical cumulative distribution functions. Moreover, Figures S5c and S5d show the KL-divergences to truth and runtimes, respectively. It seems that the KL divergence to the truth may converge more slowly for `sequence_alignment` sequences rather than CDR3s, although the approximate procedure seems to outperform the full distribution for a slightly larger range of ε values (i.e. until ε nears 10^{-6}).

Next we investigate the effect of dataset size on the performance of Algorithm 2. For sample sizes $n \in \{\exp(6), \dots, \exp(10)\}$, we subsample `p_f1` without replacement to n sequences and compute the pairwise distance distribution of CDR3 sequences for the full subsampled dataset as well as those given by tolerances $\varepsilon \in \{0.1, ..., 10^{-5}\}$. We perform this experiment 5 times for each n.

Figures S6a, S6b, and S6c display boxplots of the KL-divergence to truth, runtime, and time efficiency, respectively. There is not an obvious trend in KL divergence to truth for a given tolerance as sample size

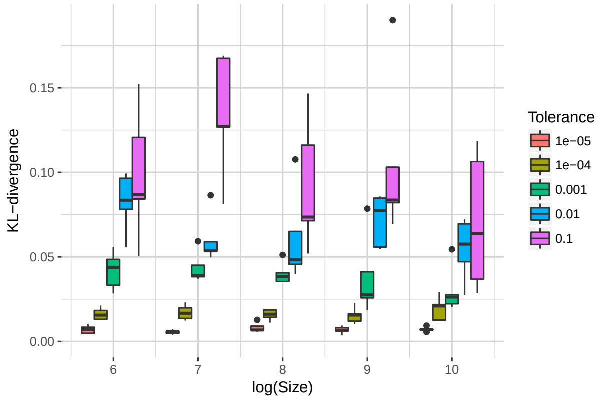

(a) KL-divergence to true nearest neighbor distribution by tolerance and log(size) of dataset, taken over 10 trials of the algorithm.

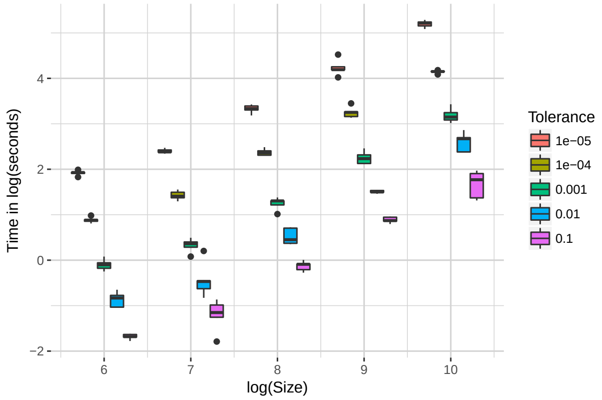

(b) Time complexity of the approximate nearest neighbor distribution by tolerance and log(size) of dataset, taken over 10 trials of the algorithm.

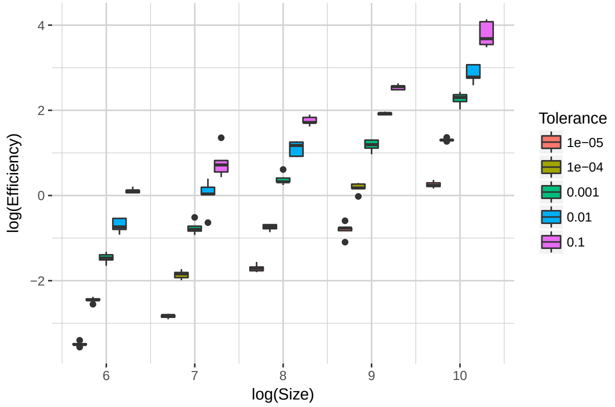

(c) KL-divergence to true nearest neighbor distribution by tolerance and log(size) of dataset, taken over 10 trials of the algorithm.

Figure S6: Performance of Algorithm 2 by sample size and tolerance applied to the nearest neighbor distribution of CDR3nt sequences.

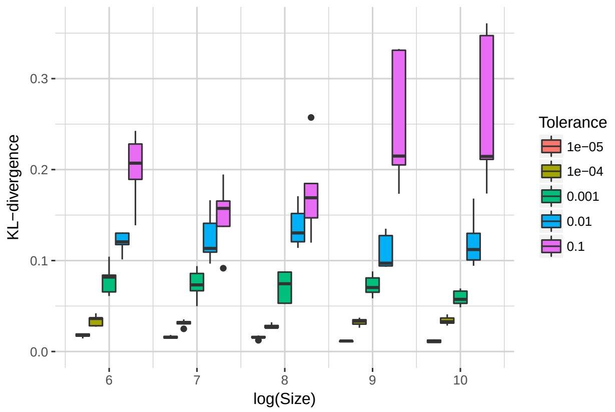

(a) KL-divergence to true nearest neighbor distribution by tolerance and log(size) of dataset, taken over 10 trials of the algorithm.

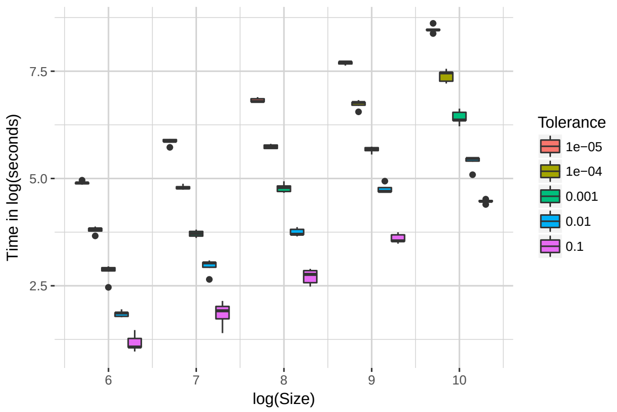

(b) Time complexity of the approximate nearest neighbor distribution by tolerance and log(size) of dataset, taken over 10 trials of the algorithm.

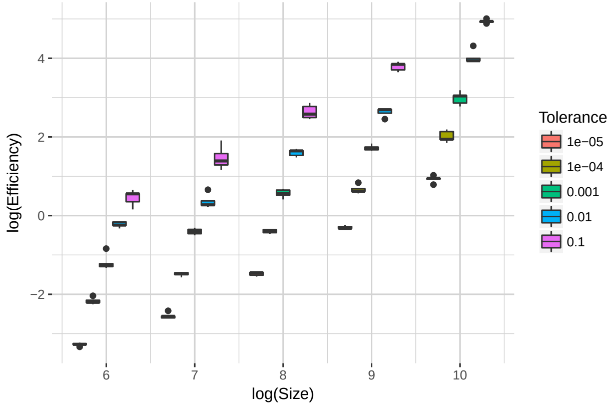

(c) KL-divergence to true nearest neighbor distribution by tolerance and log(size) of dataset, taken over 10 trials of the algorithm.

Figure S7: Performance of Algorithm 2 by sample size and tolerance applied to the nearest neighbor distribution of pairwise-aligned VDJ sequences.

increases, although the variability is higher for high tolerances. As expected, runtime increases as tolerance decreases, and also increases with the size of the dataset. This is reasonable since each batch iteration of Algorithm 2 must compute the nearest neighbor distance from each sequence in batch B to the full repertoire R, which certainly increases in time complexity as R increases.

Next we look at the efficiency relative to computing the full distribution as defined in Equation 1. Examining the boxplots near $y = 0$ by log(size), we see that for a dataset of size $\exp(k)$, we would need a tolerance of at least $\frac{1}{10^{k-4}}$. For example, for log(size) $= 6$, we see that tolerances higher than $0.01 = \frac{1}{100} = \frac{1}{10^{6-4}}$ would on average yield an efficiency greater than one. This suggests that, for a dataset with n CDR3 sequences, a sensible rule of thumb would be to choose $\varepsilon > \frac{1}{10^{k-4}} = \frac{1}{10^{\log(n)-4}}$. This will of course be more or less appropriate for a given dataset depending on the nature of the repertoire from which it was sampled.

Finally, we perfrom the same experiment but using `sequence_alignment` sequences for the nearest neighbor distance distribution. Figures S7a, S7b, and S7c display boxplots of the KL-divergence to truth, runtime, and time efficiency, respectively. There is evidence of a positive trend of the KL-divergence as sample size increases for $\varepsilon = 0.1$, although this trend seems to diminish for each other tolerance. Runtimes increase with given sample size and tolerance, and are generally higher than they are for CDR3 sequences as expected. It turns out that the efficiencies follow the same rule of thumb we derived for the CDR3 sequence situation. In particular, choosing $\varepsilon > \frac{1}{10^{k-4}} = \frac{1}{10^{\log(n)-4}}$ will on average lead to an increase in efficiency with respect to

the full distribution for sequence_alignment sequences as well as CDR3 sequences. While this may depend on the dataset in question, we recommend this as a good point of reference for general use.

The user should use these results as well as problem-specific considerations when deciding whether or not to use Algorithm 2 instead of computing the full distribution, and if so, which tolerance to use. By default, sumrep retrieves the approximate rather than full nearest neighbor distribution, and uses $\varepsilon = 10^{-4}$ unless otherwise modified.

Appendix C: Multinomial lasso path plots

Figure S8 displays the lasso path plots which illustrate the coefficient values of each response vector utilized in Algorithm 3. Figure S8a shows paths for IGoR annotations of six TRB datasets from [7], and Figure S8b shows paths for partis annotations for six IGH datasets from [21].

Appendix D: Model validation analysis workflows

Figure S9a illustrates the IGoR model validation workflow. We employ IgBLAST to obtain CDR3 sequences for the observed sequences, which IGoR only outputs for generated sequences. Moreover, because we fit IGoR models on predominantly productive TRB sequences, we consider only IGoR-generated sequences whose V and J segments are in-frame.

Figure S9b illustrates the partis model validation workflow as described in the Methods section. We first run partis partition on each fasta file of IGH sequence reads to obtain annotations for each sequence, as well as a directory containing model parameters for inference and simulation. We can then run partis simulate with these model parameters as input to generate a synthetic datset of IGH annotations. We subsample both the experimental and simulated annotation datasets to unique clones. Then, we compare each IGH-relevant summary for the two resultant annotations datasets, yielding a divergence value for each summary.

Appendix E: Comparison of summary scores using IgBLAST annotations

Recall that for the standard partis model validation procedure, partis is used for both inference as well as simulation. Here we examine the influence of using the same tool for inference and simulation by using IgBLAST for inference, and comparing the annotations dataset output from IgBLAST to the corresponding simulations from partis. The workflow for this procedure is displayed in Figure S10, which is essentially the diagram in Figure S9b with an additional path describing the IgBLAST/Change-O pipeline. Change-O was used to parse the IgBLAST output, as well as partition the sequences into inferred clonal families [15].

Figure S11a shows the LRAD-data scores by summary when using IgBLAST for annotation and partis for simulation. Figure S11b shows the difference of each score in Figure 9a and each score in Figure S11a. Frequency polygons of summary distributions of three pairs of IgBLAST-annotated and partis-simulated datasets are shown in Figure S12. The plots show a high level of agreement for most summaries, with all but six of them differing by less than one units, and a strong majority of them close to zero. Where differences arise, this is likely the result of differences in how partis and IgBLAST perform annotations. For example, we see that the insertion length distributions highly disagree in scores. This is at least partially attributable to the star-tree assumption on which partis operates, which is prone to overestimate insertion lengths in an effort to better estimate the ultimate naive sequence. Indeed, examining the VD insertion length distribution shows that IgBLAST tends to assign a similar distribution to each dataset, whereas partis leads to more variable distributions with right skew due to the star-tree assumption. Moreover, if IgBLAST tends to assign a similar insertion length distribution to every dataset, then this will make it difficult for a simulator designed to match particular insertion lengths distributions to behave more like the IgBLAST distributions. Thus, inherent differences in annotation tools will certainly lead to differences in summary scores, regardless of how accurate either tool is. Hence, it is important to understand that a given annotations-based summary should be considered in the context of the tool which provided annotations, and not as a ground-truth summary of the actual gene usage/indel statistics.

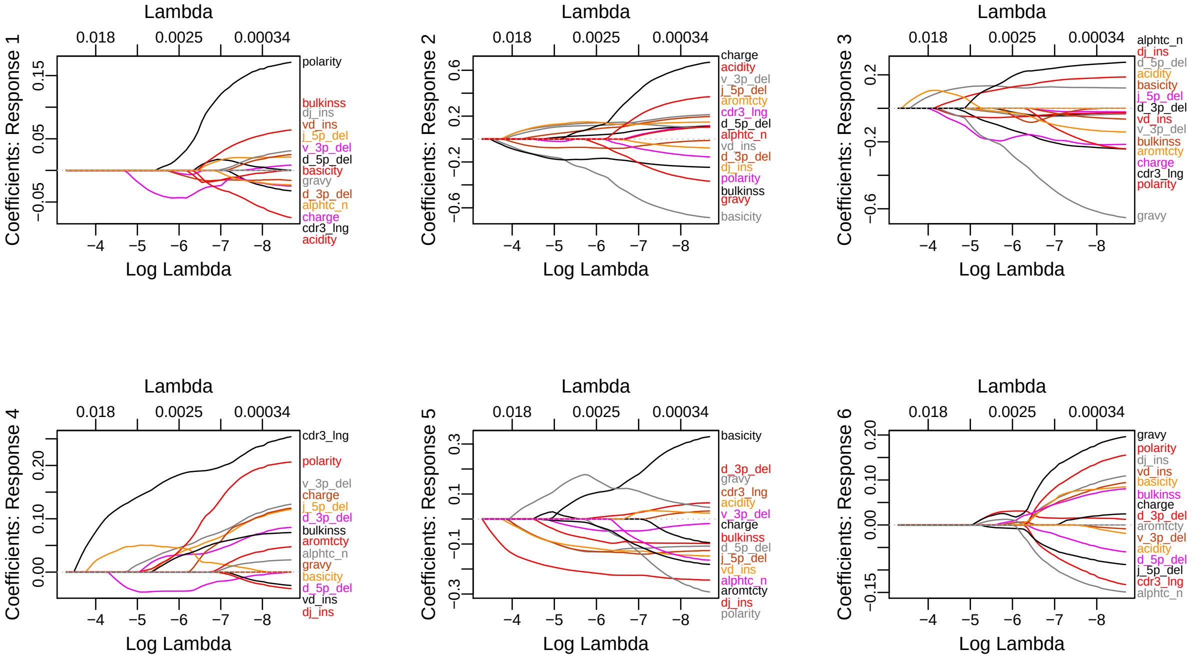

(a) Lasso paths for six `IGoR`-annotated Britanova datasets.

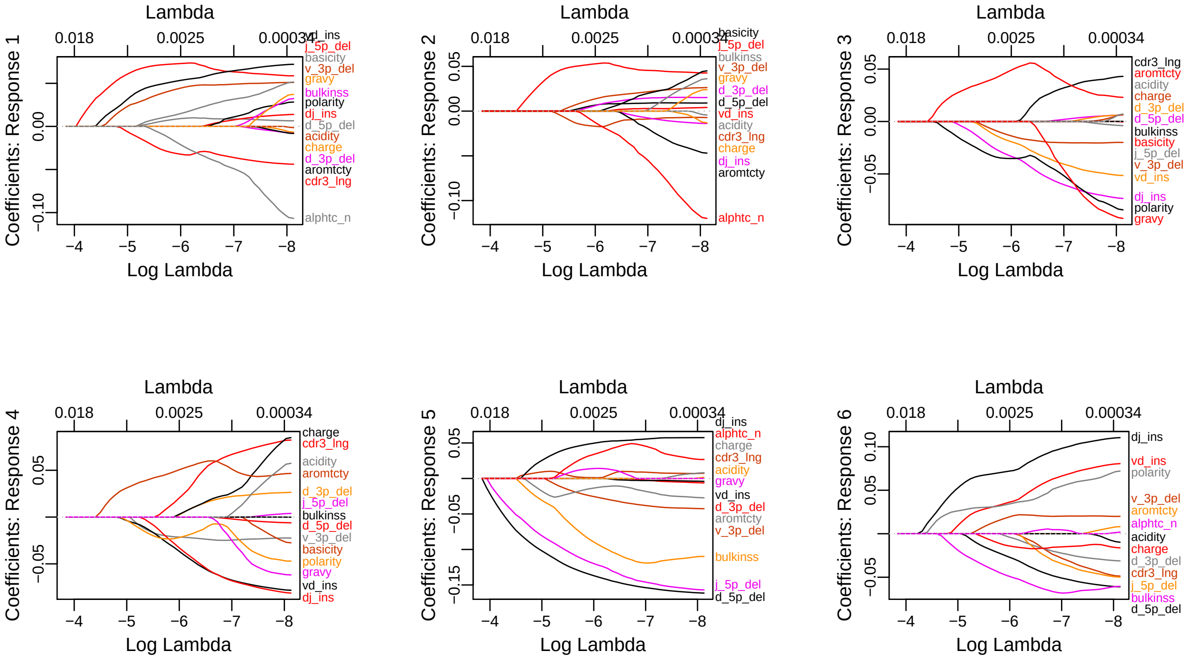

(b) Lasso paths for six `partis`-annotated Laserson datasets.

Figure S8: Multinomial lasso paths of summary coefficients by dataset identity.

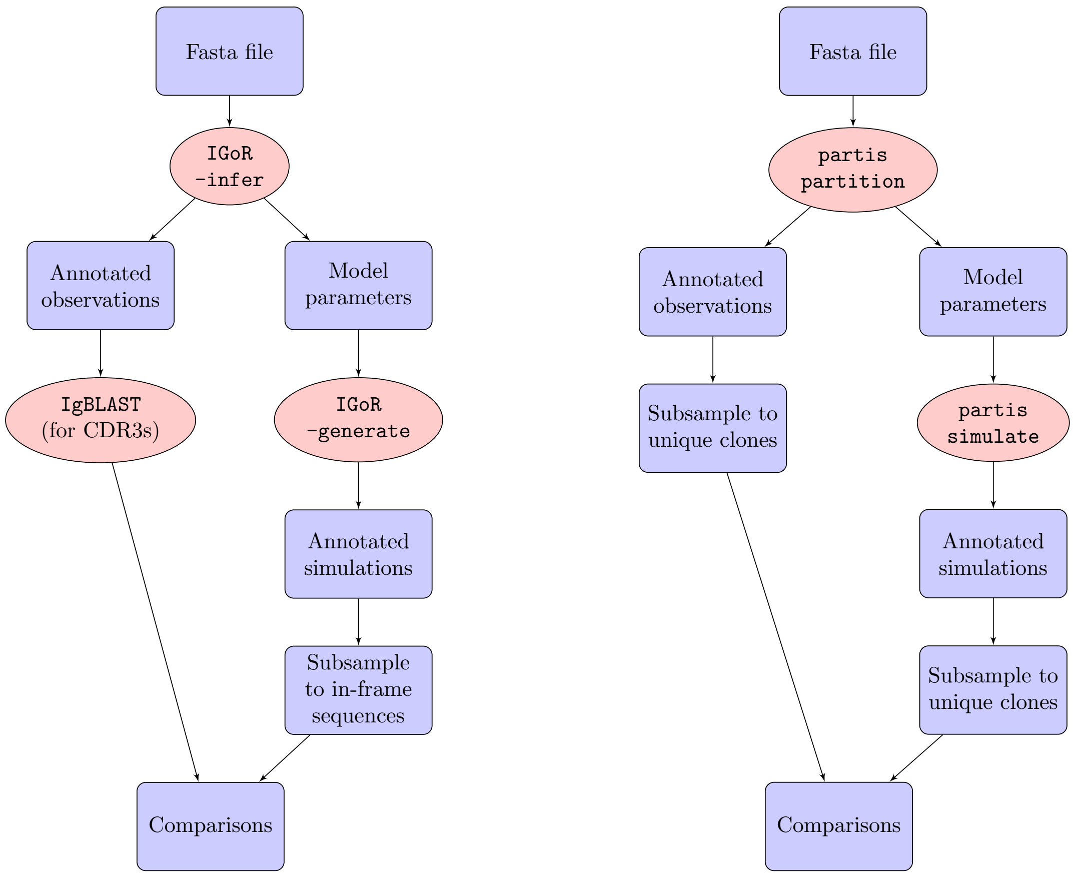

(a) Workflow for comparing a given observed repertoire dataset to an example simulated dataset via `IGoR`.

(b) Workflow for comparing a given observed repertoire dataset to an example simulated dataset via `partis`.

Figure S9: Workflow diagrams for the `IGoR` and `partis` model validation analyses.

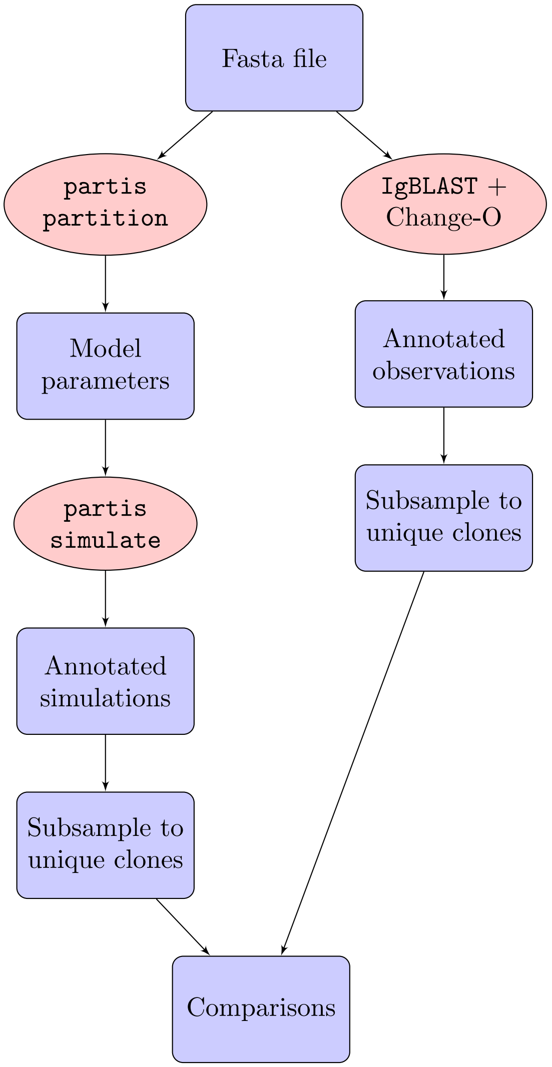

Figure S10: Workflow diagram for `partis` model validation when comparing `partis` and `IgBLAST` annotations to partis simulations

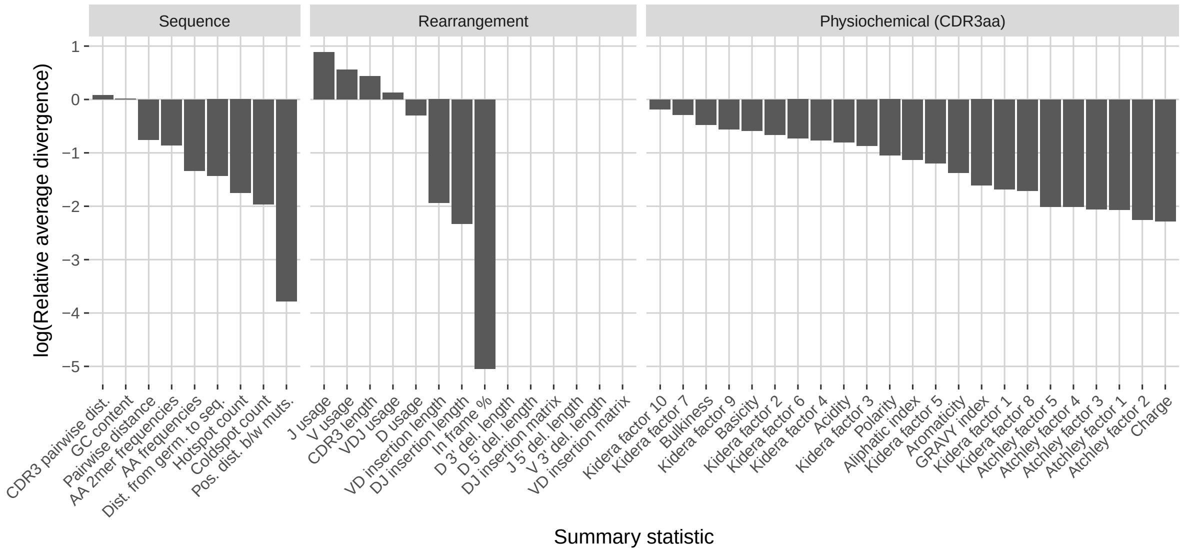

(a) Comparing divergences for `IgBLAST` annotations and `partis` simulations based on the same individual observed repertoires. We use the default germline databases in `IgBLAST` for consistency.

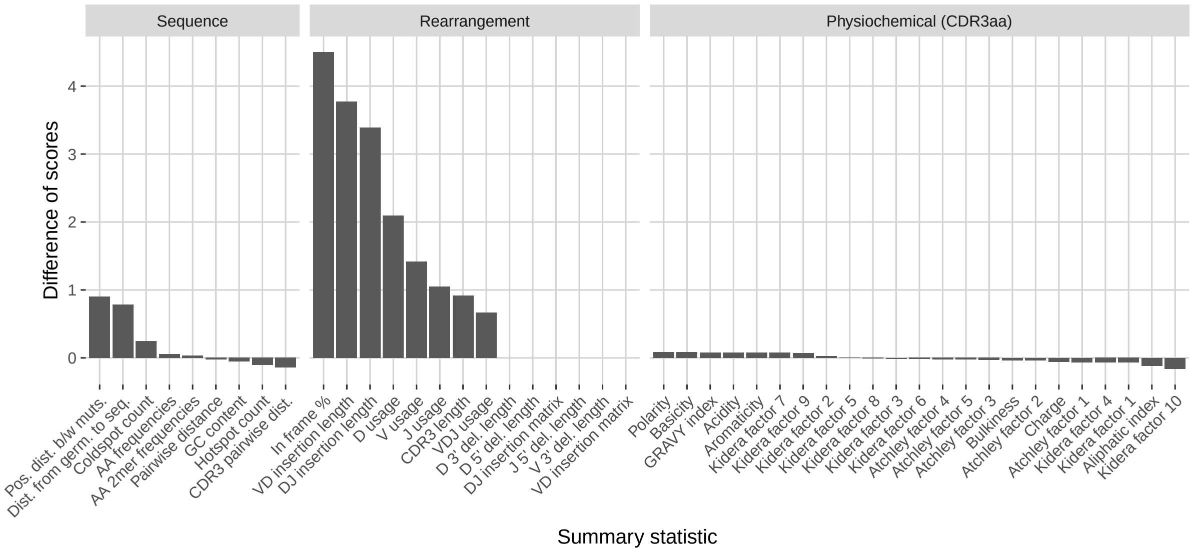

(b) Difference in LRAD values when using `IgBLAST` versus `partis` for annotations.

Figure S11: Summary scores for each statistic in the `partis` model validation experiment when comparing `partis` simulations to `IgBLAST` annotations. IMGT IGH germline databases were used during inference for both tools. In both plots, a high score indicates a well-replicated statistic by the simulations with respect to their corresponding experimental repertoires of productive IGH sequences. Summaries without a score are not readily available from AIRR-formatted `IgBLAST` output.

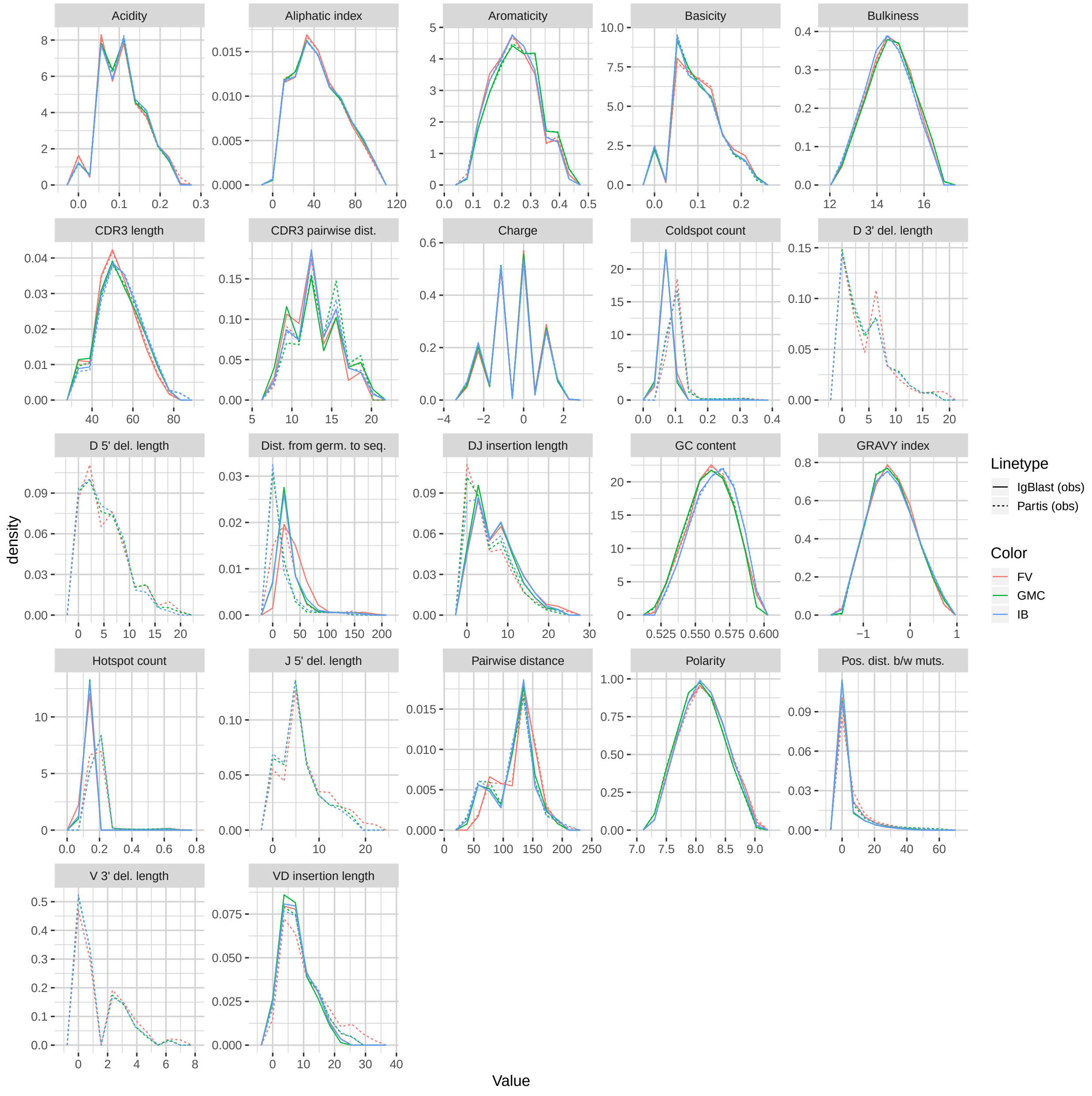

Figure S12: Summary distribution frequency polygons of `partis` versus `IgBLAST` annotations of experimental datasets from three donors at time point −1h.
